# A multi-agent model to study epidemic spreading and vaccination strategies in an urban-like environment

**DOI:** 10.1007/s41109-020-00299-7

**Published:** 2020-09-22

**Authors:** Matthieu Nadini, Lorenzo Zino, Alessandro Rizzo, Maurizio Porfiri

**Affiliations:** 1 Department of Mechanical and Aerospace Engineering, New York University Tandon School of Engineering, Brooklyn, 11201 USA; 2 Faculty of Science and Engineering, University of Groningen, Groningen, 9747 AG The Netherlands; 3 Dipartimento di Elettronica e Telecomunicazioni, Politecnico di Torino, Torino, 10129 Italy; 4 Office of Innovation, New York University Tandon School of Engineering, New York, 11201 USA; 5 Department of Biomedical Engineering, New York University Tandon School of Engineering, Brooklyn, 11201 USA

**Keywords:** Agent-based model, core-periphery structure, Epidemics, Mobility, Temporal network

## Abstract

Worldwide urbanization calls for a deeper understanding of epidemic spreading within urban environments. Here, we tackle this problem through an agent-based model, in which agents move in a two-dimensional physical space and interact according to proximity criteria. The planar space comprises several locations, which represent bounded regions of the urban space. Based on empirical evidence, we consider locations of different density and place them in a core-periphery structure, with higher density in the central areas and lower density in the peripheral ones. Each agent is assigned to a base location, which represents where their home is. Through analytical tools and numerical techniques, we study the formation mechanism of the network of contacts, which is characterized by the emergence of heterogeneous interaction patterns. We put forward an extensive simulation campaign to analyze the onset and evolution of contagious diseases spreading in the urban environment. Interestingly, we find that, in the presence of a core-periphery structure, the diffusion of the disease is not affected by the time agents spend inside their base location before leaving it, but it is influenced by their motion outside their base location: a strong tendency to return to the base location favors the spreading of the disease. A simplified one-dimensional version of the model is examined to gain analytical insight into the spreading process and support our numerical findings. Finally, we investigate the effectiveness of vaccination campaigns, supporting the intuition that vaccination in central and dense areas should be prioritized.

## Introduction

The number of people living in urban areas has already exceeded 4 billions and it is estimated to reach 7 billions by 2050 (Ritchie and Roser 2020). Global urbanization poses new challenges in different sectors, ranging from transportation to energy supply, environmental degradation, and healthcare (Cohen 2006). Among these challenges, understanding how urban environments shape the evolution of epidemic outbreaks and designing effective containment strategies have recently drawn considerable attention from researchers and media. Paradigmatic are the examples of recent outbreaks, such as the 2003 SARS (Smith 2006), 2012 MERS (de Groot et al. 2013), and the ongoing COVID-19 (Chang et al. 2020; Chinazzi et al. 2020; Ferguson et al. 2020).

Analyzing how diseases spread within urban environments has been the topic of various experimental and theoretical studies (Eubank et al. 2004; Satterthwaite 2007; Alirol et al. 2011; Neiderud 2015; Telle et al. 2016; Massaro et al. 2019). Experimental studies have offered a detailed analysis of urban environments (Satterthwaite 2007; Neiderud 2015), suggesting specific preventive measures for both urban residents and travelers (Alirol et al. 2011). Theoretical studies have provided insights on how to contain outbreaks (Eubank et al. 2004), as well on possible key drivers of contagion, such as the role of human mobility patterns (Massaro et al. 2019) and socio-economical risk factors (Telle et al. 2016).

Despite the importance of urban environments in the global diffusion of diseases (Brockmann and Helbing 2013), how epidemic outbreaks unfold therein is yet to be fully elucidated. Some attempts to mathematically describe the diffusion of diseases within and among cities can be found in metapopulation models (Colizza and Vespignani 2007; Colizza and Vespignani 2008; Liu et al. 2013). In these models, a fixed network of spatial localities is used to model the mobility patterns between cities, where homogeneously-mixed populations are affected by the epidemic. While metapopulation models can be, at least partially, tackled through analytical methods (Colizza and Vespignani 2007; Colizza and Vespignani 2008; Liu et al. 2013), considerable experimental evidence challenges the assumption of homogeneously-mixed populations, which could yield misleading estimates of the extent of epidemic outbreaks (Pastor-Satorras et al. 2015).

On the other side of the spectrum of epidemic models, agent-based models (Eubank et al. 2004; Degli Atti et al. 2008; Gilbert 2008) constitute a valuable framework to offer a realistic description of how diseases diffuse within urban environments. Currently, this class of models is being leveraged to predict the diffusion of the COVID-19 (Chang et al. 2020; Ferguson et al. 2020), informing the design and implementation of timely containment measures. However, those advantageous features are accompanied by some drawbacks, including the need of mobility data and models, the use of massive computational resources when the system size scales up, and the lack of analytical techniques for model characterizations. A viable approach to agent-based modeling is based on two-dimensional representations, where agents move and interact according to proximity criteria (Frasca et al. 2006; Frasca et al. 2008; Zhou and Liu 2009; Buscarino et al. 2010; Yang et al. 2012; Buscarino et al. 2014; Huang et al. 2016; Peng et al. 2019). As a first approximation, the motion of the agents can be described according to a random walk with sporadic long range jumps (Frasca et al. 2006). Building on this approximation, it is possible to include realistic features such as nonhomogeneous infection rates (Buscarino et al. 2014) and heterogeneous radii of interaction (Huang et al. 2016; Peng et al. 2019). Much work is needed, however, to fully capture and describe realistic patterns of human mobility, which are shaped by the complex structure of urban environments (Alessandretti et al. 2017).

Here, we contribute to the field of agent-based modeling by presenting a two-dimensional model that is capable of reproducing a spatially inhomogeneous urban-like environment, in which a heterogeneous population follows realistic rules of mobility. Inspired by previous theoretical studies (Huang et al. 2016; Peng et al. 2019), we assume that agents have a heterogeneous radius of interaction, which accounts for variations among individuals in their involvement in social behavior and activities.

We consider a urban-like environment composed of multiple locations, each of them representing a well-defined region of the urban space (that is, a neighborhood of a city). Through this spatial organization, our model is able to encapsulate two key features of urban environments. First, it can reproduce typical core-periphery structures, where central regions are more densely populated than peripheral ones (Makse et al. 1998; De Nadai et al. 2016). Second, it allows to mimic the inhomogeneity in movement patterns of humans, where people tend to spend most of their time in a few neighborhoods — for example, experimental studies suggest that individuals spend most time either at home or at work, while only sporadically visiting other neighborhoods (Brasche and Bischof 2005; Schweizer et al. 2007; Matz et al. 2015).

To reproduce realistic conditions for agents’ movement patterns, we posit two different mobility schemes, applied within and outside the agents’ base location (that is, where their home is). While the homogeneous mixing assumption seems reasonable within the agents’ base location, we assume that agents tend to move outside of their base location following a gravity model and a biased random walk. Hence, agents are more likely to explore regions close to their base location rather than remotely-located regions (Brasche and Bischof 2005; Schweizer et al. 2007; Matz et al. 2015). From this mobility pattern, we construct a network of contacts, whose topology is examined in this study. Through some mathematical derivations and numerical simulations, we seek to identify analogies between the proposed agent-based model and existing temporal network approaches, where spatial mobility is lumped into nodal parameters (Perra et al. 2012; Zino et al. 2016; Zino et al. 2018; Nadini et al. 2018; Nadini et al. 2020).

We adopt the proposed framework to study how urban-like environments shape the diffusion of infectious diseases, using the illustrative epidemic models with the possibility of reinfection (susceptible–infected–susceptible, or SIS) or permanent removal (susceptible–infected–removed, or SIR) (Keeling and Rohani 2011). Our results confirm the intuition that agents’ density plays a critical role on the diffusion of both SIS and SIR processes. In the limit case where the entire urban area consists of one location, agents that move outside the location only seldom interact with other agents, thereby hindering the contagion process.

In the more realistic scenario of a core-periphery structure with multiple locations, we unexpectedly find that the time spent by agents in their base location does not influence the endemic prevalence in the SIS model and the epidemic size in the SIR model, which are measures of the overall fraction of population that is affected by the disease. A possible explanation for this counterintuitive phenomenon may be found in the agents’ mobility rules. In fact, commuting patterns that bring agents from central areas to peripheral ones may yield a reduction in the diffusion in the central areas. Contrarily, commuting patterns from peripheral to central areas lead to the opposite effects. To detail the working principles of this unexpected result, we present a minimalistic one-dimensional version of the model, which is amenable to a complete analytical treatment, thereby offering some preliminary analytical insight into the role of model parameters on epidemic spreading.

We also explore the interplay between the agents’ radius of interaction and their positioning in the core-periphery structure. We find that when agents’ with larger radii are assigned to the less dense and peripheral locations, then the endemic prevalence (in the SIS model) and the epidemic size (in the SIR model) strongly decrease with respect to a random assignment. Moreover, when agents’ with larger radii are assigned to denser (and central) locations, the fraction of population affected by the disease is not sensibly increased. Hence, our results support the intuition that more central areas are the crossroads of individuals commuting in a city and are critical for the spread of diseases.

Finally, we numerically analyze the effect of targeted vaccination strategies, which consist of immunizing a portion of the population in a specific location, prior to the disease onset. Consistent with the intuition that central locations play a key role on the spread of epidemic diseases, we find that the best strategy is to prioritize the vaccination of agents belonging to central urban areas.

The rest of the manuscript is organized as follows. In Table 1, we summarize the notation and the nomenclature used throughout the paper. In “Model”, we introduce the model of agents’ mobility. In “Temporal network of contacts”, we describe and analyze the temporal network formation mechanism. In “Epidemic processes”, we analytically and numerically study the spread of epidemic processes and compare several vaccination strategies. In “Discussion and conclusion” sections, we discuss our main findings and propose further research directions.

**Table 1:** Nomenclature of the variables and notation used in the paper

Symbol	Description
$\mathcal {V}$	Set of all agents
$\mathcal {L}$	Set of all locations
*N*	Number of agents in the system
*L*	Number of locations in the system
*n*	Number of agents in each location
*D*	Side of the square planar space
*T*	Total number of discrete time-steps in the observation window
*t*	Index for discrete time instants
*ℓ*	Index for the locations in the system
*i*,*j*	Indices for the agents in the system
*ρ*_*ℓ*_	Density of location *ℓ* in the system
*β*(*i*)	Function that maps agent *i* to its base location
*Ω*_*ℓ*_	Region of space occupied by location *ℓ*
*x*_*i*_(*t*),*y*_*i*_(*t*)	Position occupied by agent *i* in the planar space at time *t*
*θ*_*i*_(*t*)	Direction of the motion of agent *i* at time *t*
*Φ*_*i*_(*t*)	Angle of the direction of the shortest path from *x*_*i*_(*t*),*y*_*i*_(*t*) to *Ω*_*β*(*i*)_
*Δθ*_*it*_	Angle drawn uniformly at random in [0,2*π*) for agent *i* at time *t*
*v*	Velocity of agents outside their base location
*α*	Randomness in the agents’ motion toward their base location
*p*	Probability of jumping outside the base location
*P*_jump_(*d*)	Probability of jumping at a distance *d* from the base location
*q*_in_	Probability of being inside the base location
*q*_out_	Probability of being outside the base location
*q*_*ℓ*_	Probability of being inside *Ω*_*ℓ*_
*q*_out,*d*_	Probability of being at a distance *d* from the closest location
*Ψ*_in_,*Ψ*_out_,*Ψ*_*d*_	Probability that agents are inside, outside, and at a distance *d*
	from their base location
*P*(*Σ*)	Probability density function of locations’ radii
*γ*	Exponents of the power law distribution of locations’ radii
*Σ*_min_,*Σ*_max_	Lower and higher cut-off of the distribution of locations’ radii
*G*(*σ*)	Probability density function of agents’ radii of interaction
*ω*	Exponents of the power law distribution of radii of interaction
*σ*_min_,*σ*_max_	Lower and higher cut-off of the distribution of radii of interaction
*k*_*i*_	Degree of agent *i*
*λ*	Infection probability per contact
*μ*	Recovery probability per unit time
*s*(*t*),*i*(*t*),*r*(*t*)	Fraction of susceptible, infected, and recovered agents in the system
*s*_*ℓ*_(*t*),*i*_*ℓ*_(*t*),*r*_*ℓ*_(*t*)	Fraction of susceptible, infected, and recovered agents in *Ω*_*ℓ*_
*s*_out,*d*_(*t*),*i*_out,*d*_(*t*),*r*_out,*d*_(*t*)	Fraction of susceptible, infected, and recovered agents at distance *d*
	from the closest location
*Λ*_*ℓ*_(*t*)	Contagion probability in *Ω*_*ℓ*_
*Λ*_out,*d*_(*t*)	Contagion probability at a distance *d* from the closest location
〈·〉	Statistical average of the quantity “ ·”
E [·]	Expected value of the quantity “ ·”
*P*[·]	Probability of an event “ ·”

## Model

We consider *N*≥1 agents, labeled by positive integers $\mathcal V:=\{1, \dots, N\}$. Agents move in a square planar space with side length *D*>0 and with periodic boundary conditions (Frasca et al. 2006), that is, when an agent exits through one side of the square planar space, it re-appears on the opposite side. The position of agent $i\in \mathcal V$ at the discrete time $t\in \mathbb Z_{\geq 0}$ in a Cartesian reference frame is denoted by (*x*_*i*_(*t*),*y*_*i*_(*t*))∈[0,*D*]×[0,*D*].

### Urban-like environment

We deploy the *N* agents over *L* locations, each of them representing a bounded portion of the square space. The set of all locations is $\mathcal {L} = \{1, \dots, L \}$ and each location $\ell \in \mathcal {L}$ occupies a convex region of the planar space *Ω*_*ℓ*_⊂[0,*D*]×[0,*D*] with area *A*_*ℓ*_. We assume that all the locations are mutually disjoint and we order them in ascending order according to their area, that is, $A_{1}\leq \dots \leq A_{L}$. We hypothesize that *A*_*L*_≪*D*^2^, that is, each location is much smaller than the whole square space. Each agent is assigned a specific base location (that is, their home) according to a map: $\beta :\mathcal V\longrightarrow \mathcal {L}$; we assume that each base location is associated with the same number of agents, *n*=*N*/*L*.^1^ As a result, the density of agents assigned to location *ℓ*,

1 $$ \rho_{\ell} := \frac{n}{A_{\ell}}\,,   $$

varies with the location. Also, locations are sorted in descending order of density, that is $\rho _{1}\geq \dots \geq \rho _{L}$.

For simplicity, in the numerical simulations implemented throughout this paper, the convex regions are taken as circles with nondecreasing radii $\Sigma _{1}\leq \dots \leq \Sigma _{L}$. Inspired by empirical and theoretical studies (Witten Jr and Sander 1981; Vicsek 1992; Makse et al. 1998; De Nadai et al. 2016), radii of the locations are extracted from a power law distribution with probability density function *P*(*Σ*)∝*Σ*^−*γ*^, with bilateral cutoffs such that *Σ*_*ℓ*_∈[*Σ*_min_,*Σ*_max_], for any $\ell \in \mathcal {L}$. The upper bound guarantees that all locations fit in the square, and the lower bound sets a maximum to the locations’ density. Since the radii are power law distributed with exponent −*γ*, the areas of the locations are also power law distributed with exponent −2*γ* and cutoffs such that $A_{\ell }\in \left [\pi {\Sigma }^{2}_{\text {min}},\pi {\Sigma }^{2}_{\text {max}}\right ]$.

**Fig. 1:**
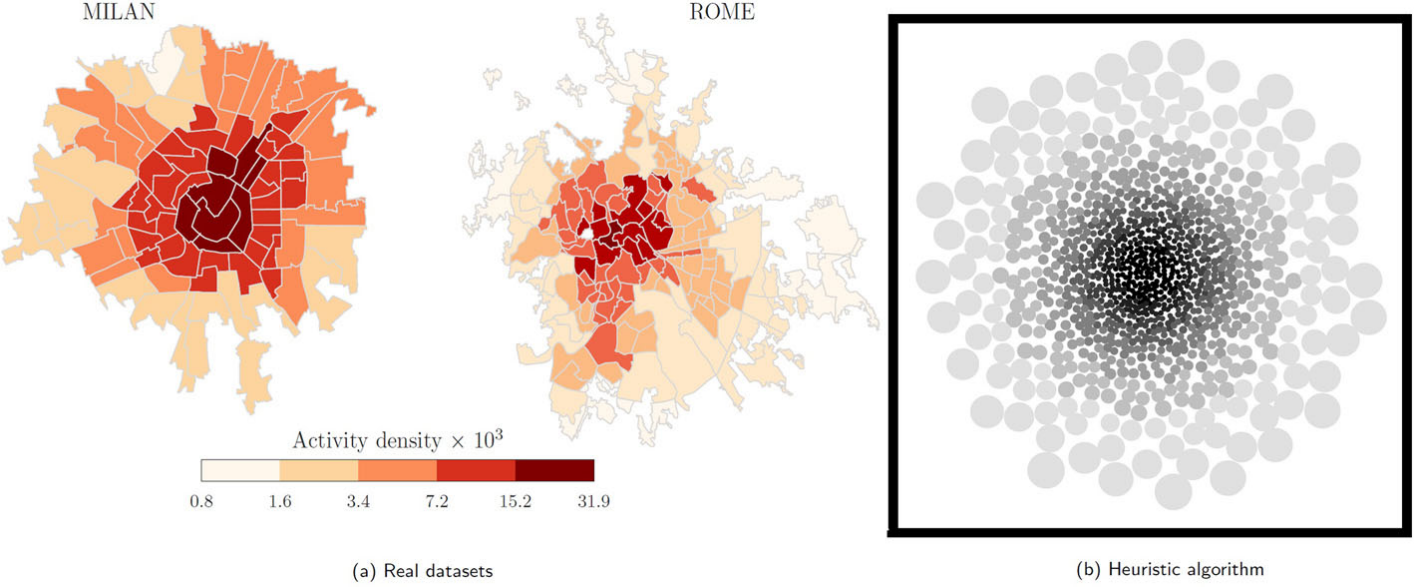
Qualitative comparison between real datasets from an experimental study (De Nadai et al. 2016), and the output of our algorithm. **a** Experimental results about human digital activity density in the cities of Milan and Rome, Italy. The highest density is registered in central areas, while lower densities are observed in peripheral ones. **b** Using our algorithm, we generate *L*=1,000 circular locations distributed in rings of decreasing densities. The first few rings contain the denser locations (darker central regions) and may parallel the city center of a urban environment, while the outer rings are less dense and represent peripheral areas (light gray regions). Source of **a**: (De Nadai et al. 2016). Parameters used to generate **b**: *D*=1,000, *Σ*_min_=3, *Σ*_max_=30, and *γ*=2.1. Details of the generative algorithm used are available in Appendix A

Empirical studies on urban environments suggest that cities are constructed according to a core-periphery structure, whereby locations with smaller areas and denser population are located in their center, while locations with larger areas and sparser population pertain to peripheral areas (Makse et al. 1998; De Nadai et al. 2016), as shown in Fig. 1a. We implement a heuristic algorithm to generate a locations’ layout according to a core-periphery structure and qualitatively reproduce empirical results. Figure 1b shows the output generated by our algorithm, whose structure is qualitatively consistent with the empirical observations reported in Fig. 1a. Details of the algorithm used to create such a core-periphery structure are presented in Appendix A.

At the present time, the technical literature has yet to empirically study the relationship between the agents’ radius of interaction and the density of their base location. In this paper, we explore different scenarios aiming at offering a first theoretical understanding of the impact of this potential relationship on the evolution of disease processes. Unless otherwise specified, we consider that the *n* members of each location are randomly chosen, independently of their radius of interaction. We also examine the cases in which there is a correlation (positive or negative) between the agents’ radius of interaction and the density in their base location: a positive correlation means that agents with larger radius are assigned to denser (central) locations, while a negative correlation identifies the case in which agents with larger radius are placed in the less dense (peripheral) locations.

### Law of motion

Agents’ positions evolve according to a discrete-time dynamics. Hence, their positions are updated at each discrete time-step $t\in \mathbb Z_{\geq 0}$. The law of motion of the generic agent *i* depends on whether it is outside or inside its base location $\beta (i) \in \mathcal {L}$. If agent $i\in \mathcal { V}$ is outside its base location, that is, (*x*_*i*_(*t*),*y*_*i*_(*t*))∉*Ω*_*β*(*i*)_, it performs a biased random walk toward its base location;^2^ on the contrary, if it is inside its base location, it can move to a random position (within its base location), or exit according to a probabilistic mechanism.

Specifically, if the agent is not in its base location, then

2 $$ \left\{\begin{array}{cc} x_{i} \left(t+1\right) = x_{i}(t) + v \cos \theta_{i} (t)\,, \\ y_{i} \left(t+1 \right)= y_{i}(t) + v \sin \theta_{i}(t)\,.  \end{array}\right.  $$

Here, *v*>0 is the (constant) speed and *θ*_*i*_(*t*) is an angle, determined as the sum of two terms:

3 $$ \theta_{i}(t) :=\Phi_{i}(t)+ \alpha \Delta \theta_{it}\,.   $$

The first term, *Φ*_*i*_(*t*), represents the angle of the direction of the shortest path from (*x*_*i*_(*t*),*y*_*i*_(*t*)) to the region *Ω*_*β*(*i*)_, that is, to the agent base location. This quantity is formally defined by introducing

4 $$ \left(\bar x_{i}(t),\bar y_{i}(t)\right):=\underset{(x,y)\in \Omega_{\beta(i)}}{arg\,min} \left\{\left(x_{i}(t)-x\right)^{2}+\left(y_{i}(t)-y\right)^{2}\right\}\,,  $$

so that

5 $$ \Phi(t):=\left\{\begin{array}{ll}\arctan\frac{\bar y_{i}(t)-y_{i}(t)}{\bar x_{i}(t)-x_{i}(t)}&\text{if }\bar x_{i}(t)>x_{i}(t)\,,\\ \pi+\arctan\frac{\bar y_{i}(t)-y_{i}(t)}{\bar x_{i}(t)-x_{i}(t)}&\text{if }\bar x_{i}(t)< x_{i}(t)\,,\\ +\frac\pi2&\text{if }\bar x_{i}(t)=x_{i}(t)\text{ and }\bar y_{i}(t)>y_{i}(t)\,,\\ -\frac\pi2&\text{if }\bar x_{i}(t)=x_{i}(t)\text{ and }\bar y_{i}(t)< y_{i}(t)\,.\end{array}\right.  $$

The second term, *αΔθ*_*it*_, is modulated by *Δθ*_*it*_, which is a random variable that takes values uniformly in [−*π*,*π*] and is extracted independently at every time *t* and for every agent *i*, and by *α*∈[0,1], which is a randomness parameter that regulates how much the agents tend to deviate from the shortest path to return to their base location, when they are outside it. When *α*=1, the agent moves completely at random, while, when *α*=0, it moves along the shortest path toward its location.

**Fig. 2:**
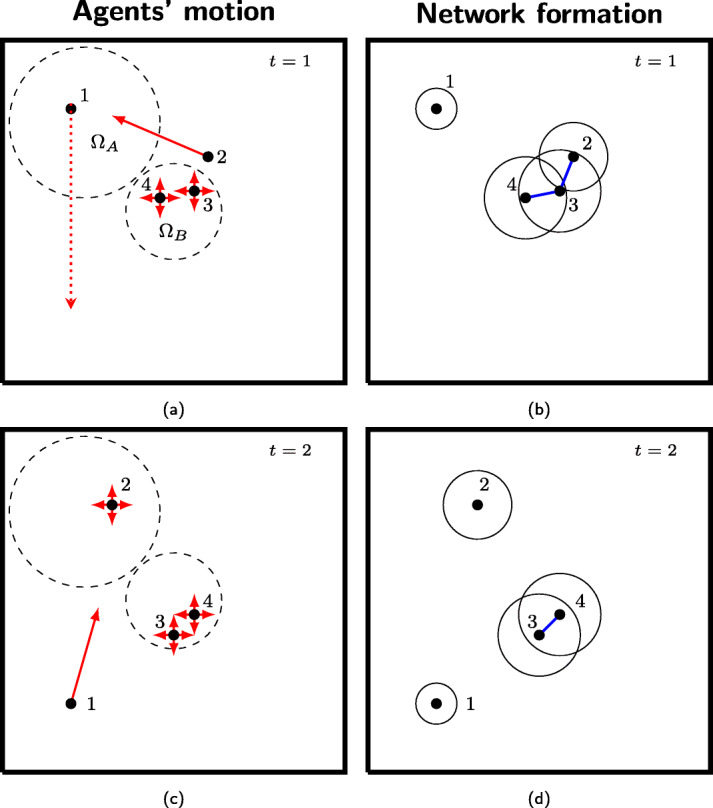
Schematic representation of two snapshots of our model with *N*=4 agents and *L*=2 locations. The entire space is delimited with solid black lines. In **a** and **c**, we illustrate the agents’ motion and the border of the locations is represented with dashed black lines. Agents 1 and 2 are assigned to location *A*:=*β*(1)=*β*(2), while agents 3 and 4 are assigned to location *B*:=*β*(3)=*β*(4). Direction and modulus of the agents’ velocity is drawn with solid red arrows. The position where agent 1 will jump is indicated with a dotted red arrow. The four arrows around an agent indicate that it will move in a random position inside its own location. In **b** and **d**, we show the temporal network formation mechanism. Agents’ radii of interaction are represented with solid circles, and undirected links are represented with solid blue lines

When the agent is in its base location, (*x*_*i*_(*t*),*y*_*i*_(*t*))∈*Ω*_*β*(*i*)_, the law of motion is defined as follows. Given a parameter *p*∈[0,1] (constant in time and equal for all agents), with probability 1−*p*, the agent moves to a position chosen uniformly at random within its base location, so that its position is completely uncorrelated with the previous one. Otherwise, with probability *p*, the agent jumps outside its base location, ending in a position of the remaining space according to a distance decay law. In particular, we assume that the distance from the border of the base location at which an agent jumps is the realization of a random variable exponentially distributed with exponent *c*>0. The corresponding probability density function *P*_*jump*_(*d*) is equal to

6 $$ P_{{jump}}(d)= ce^{-cd}\,,   $$

for *d*≥0. Hence, the expected distance at which an agent jumps is equal to 1/*c*. A sensible choice of the exponent in the law in Eq. (6) yields a typical behavior observed in many empirical studies (Levinson and El-Geneidy 2009; Boussauw et al. 2011), whereby agents tend to gravitate within and around their base location, while sporadically initiating journeys toward further locations (Liu et al. 2014). Two salient snapshots of agents’ motion are illustrated in Fig. 2a and c.

## Temporal network of contacts

Upon the mobility model, we construct the network of contacts, which is the means through which the disease is transmitted. In this vein, agents create undirected temporal links based on proximity with other agents. Specifically, agent $i\in \mathcal V$ contacts all other agents located within a circle of radius *σ*_*i*_ centered in its current position (*x*_*i*_(*t*),*y*_*i*_(*t*)). We assume that agents have heterogeneously distributed radii, extracted from a power law distribution with probability density function *G*(*σ*)∝*σ*^−*ω*^, with suitable cutoffs so that *σ*∈[*σ*_min_,*σ*_*max*_].

An undirected temporal link between two agents *i* and *j* is created when the Euclidean distance at time *t* between the position of agent *i*, (*x*_*i*_(*t*),*y*_*i*_(*t*)), and the position of agent *j*, (*x*_*j*_(*t*),*y*_*j*_(*t*)), is less than or equal to the maximum of the two radii *σ*_*i*_ and *σ*_*j*_, that is,

7 $$ \sqrt{\left(x_{i}(t) - x_{j}(t)\right)^{2} + \left(y_{i}(t) - y_{j}(t)\right)^{2}} \leq \max\left\{{\sigma}_{i}, {\sigma}_{j}\right\}\,.   $$

Figure 2b and d show two consecutive instances of the network formation process. Toward modeling of epidemics in urban environments, our model allows agents inside a location to interact with agents outside the location, see, for example, agents 2 and 3 in Fig. 2a and b.

The intricacy of the motion patterns and the nonsmooth process for generating the network of contacts hinder the analytical tractability of the model in its general formulation. However, for some cases it is possible to establish analytical insight on some model features. In Appendix B, we analyze the system in the two specific cases of: i) a free space without any location (*L*=0), and ii) when the law of motion of the agents outside their base locations is deterministic (*α*=0) and the locations are uniformly distributed in the plane. In these two cases it is possible to apply a meanfield approach in the limit of large systems (*N*→*∞*) to analytically study the number of connections generated by the agents, which represent potential paths of infection throughout the population. Therein, numerical simulations for large systems are provided to validate theoretical findings. The general case of a core-periphery structure and stochasticity in the motion out of the location is treated only through numerical simulations, in which we record all the interactions and use their time-evolution over sufficiently long time-windows (*T*≫1, where *T* is the duration of the observation) to study key topological features (average degree and clustering coefficient).

**Fig. 3:**
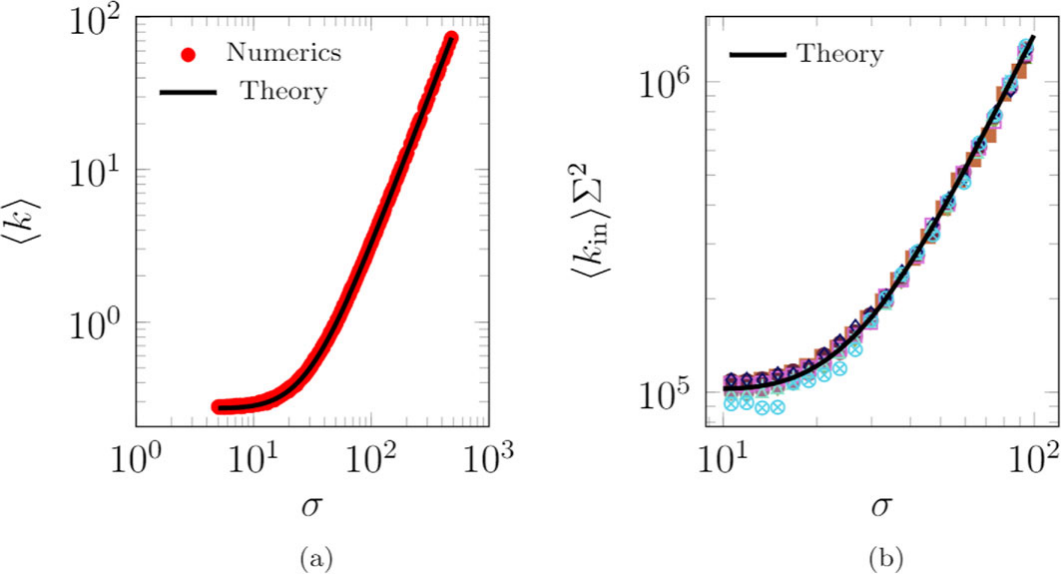
Relationship between the agents’ average degree and radius of interaction. **a** Comparison between numerical results and analytical predictions from Eq. (8), for the case without base locations. Simulation results are generated with the following parameter set: *L*=0, *D*=100,000, *σ*_min_=5, and *σ*_max_=500. For each value of *σ*_*i*_, Eq. (8) provides the expected degree, which is numerically estimated by tracking the corresponding agent in time. **b** Comparison between numerical results and analytical predictions from Eq. (9), in the case of multiple base locations, uniformly distributed in the plane. For each value of *σ*_*i*_ and *Σ*_*β*(*i*)_, Eq. (9) provides the expected degree, which is numerically estimated by tracking the corresponding agent in time. Numerical results are presented using different colors and markers, corresponding to each of the locations (numerical findings share a common trend, which is well captured by the theory). In the simulations, we use the following parameters: *L*=10, *D*=10^9^, *Σ*_min_=1,000, *Σ*_max_=10,000, *σ*_min_=10, *σ*_max_=100, *p*=0.3, and *α*=0. Agents are initially inside their base location and interactions are recorded after 100 steps to allow agents to reach a steady-state configuration. Other parameter values are *N*=10,000, *v*=500, *c*=4·10^−4^, *ω*=2.4, *γ*=2.1, and *T*=5,000

In Fig. 3a, we consider the case without locations. Our numerical results are consistent with analytical predictions in Appendix B, which are exact in the thermodynamic limit of large systems *N*→*∞*. Specifically, the expected degree of agent *i* is equal to

8 $$ \mathrm{E} \left[k_{i} \right] = \frac{{N} \pi}{D^{2}} \left(\left(1-\frac{\sigma_{i}^{1-\omega}-\sigma_{{max}}^{1-\omega}}{\sigma_{{min}}^{1-\omega}-\sigma_{{max}}^{1-\omega}}\right)\sigma^{2}_{i}+ \frac{(\omega-1)\left(\sigma_{{max}}^{3-\omega}-\sigma_{i}^{3-\omega}\right)}{(3-\omega)\left(\sigma_{{min}}^{1-\omega}-\sigma_{{max}}^{1-\omega}\right)}\right)\,,  $$

so that agents with a larger radius of interaction have a greater average degree. Note that when the agent radius is close to the minimum, that is, *σ*_*i*_≈*σ*_min_, Eq. (8) is dominated by the second summand, while when the radius is close to the maximum, that is, *σ*_*i*_≈*σ*_max_, the right-hand side of Eq. (8) scales with ${\sigma }_{i}^{2}$. Equation 8 highlights a nontrivial relationship between the expected degree of an agent and its radius of interaction, which is due to the links passively received by the agent when it is located within the radii of interaction of other agents. This relationship is different from the case of directed interactions analyzed in Huang et al. (2016); Peng et al. (2019), where *E*[*k*_*i*_] is proportional to $\sigma _{i}^{2}$.

In Fig. 3b, we examine the case of multiple locations uniformly distributed in the plane. Based on the theoretical derivations in Appendix B, we obtain the following expression for the expected number of interactions that agent *i* establishes in its own location in the thermodynamic limit of large systems *N*→*∞*:

9 $$ \mathrm{E} \left[k_{{in}, i} \right] = \frac{q^{2}_{{in}}}{\Sigma_{\beta(i)}^{2}}~(n-1) \left(\sigma^{2}_{i} + \frac{\sigma_{i}^{1-\omega}-\sigma_{{max}}^{1-\omega}}{\sigma_{{min}}^{1-\omega}-\sigma_{{max}}^{1-\omega}} \left(\frac{(\omega-1)\left(\sigma_{{max}}^{3-\omega}-\sigma_{i}^{3-\omega}\right)}{(3-\omega)\left(\sigma_{i}^{1-\omega}-\sigma_{{max}}^{1-\omega}\right)}- \sigma^{2}_{i} \right)\right)\,.   $$

We observe that E [*k*_in,*i*_] is inversely proportional to the square of the radius of location *β*(*i*), that is, $\Sigma ^{2}_{\beta (i)}$. In Fig. 3b, we multiply the numerical estimation of each agents’ average degree by the square of the radius of the corresponding location, to allow a graphical representation of the comparison between numerical estimations and analytical predictions. Numerical results in finite-size systems are in close agreement with analytical predictions of Eq. (9), which are exact in the limit of large systems.

**Fig. 4:**
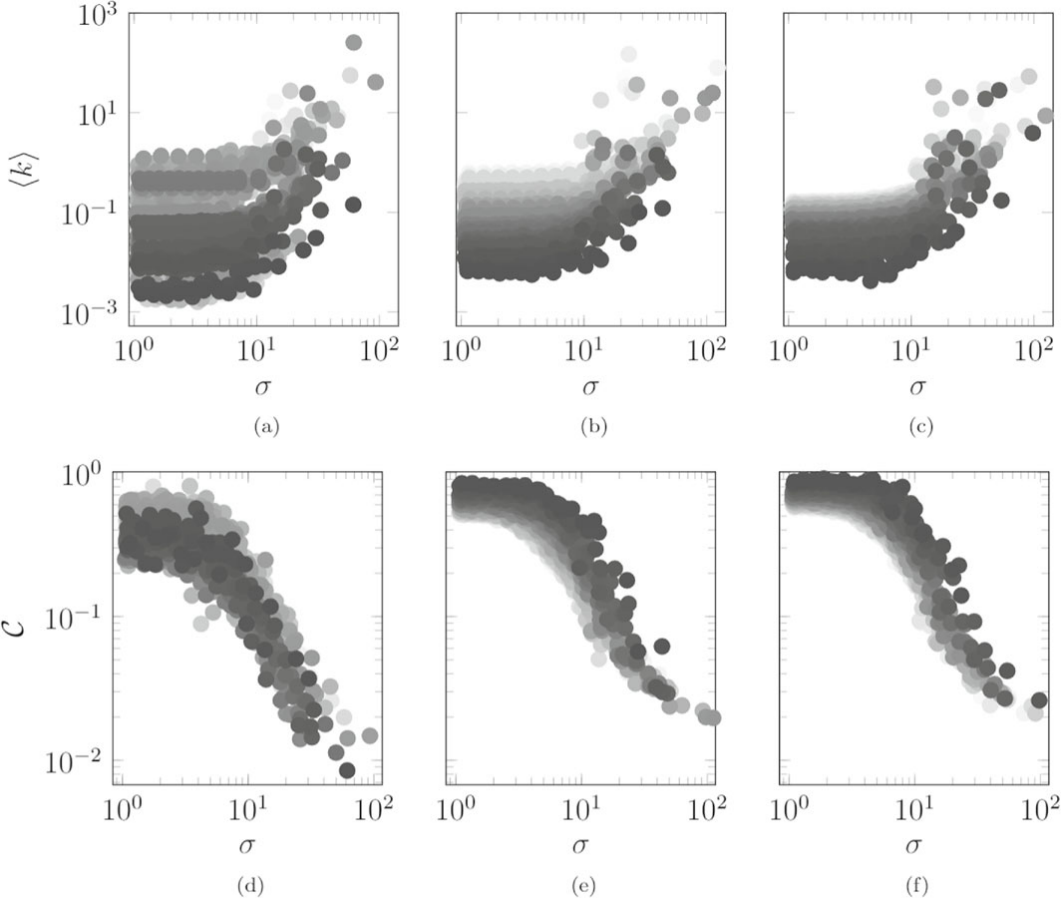
Influence of the location radius on the agents’ average degree **a**-**c** and clustering coefficient **d**-**f**, for three different parameter settings. Average degree and cluster coefficient are numerically estimated by tracking every agent in the system. Darker circles represent agents assigned to more peripheral locations, while brighter ones indicates agents belonging to more central locations. We set: **a**-**d** *p*=0.1 and *α*=0, **b**-**e** *p*=0.4 and *α*=0.2, and **c**-**f** *p*=0.8 and *α*=0.4. Agents are initially inside their base location and contacts are recorded after 100 steps to allow the agents to reach a steady-state configuration. Other parameter values are *L*=100, *N*=10,000, *D*=10^9^, *Σ*_min_=100, *Σ*_max_=10,000, *σ*_min_=1, *σ*_max_=1,000, *v*=500, *c*=4·10^−4^, *ω*=2.4, *γ*=2.1, and *T*=5,000

In order to offer insight into the influence that a core-periphery structure has on the agents’ average degree, we analyze three different scenarios. First, we study the case in which agents are strongly tied to their base location, such that they have low probability of jumping outside their base location (small *p*) and low probability of deviating from the shortest path to return to the base location, when they are outside (small randomness *α*), in Fig. 4a. Second, we examine the case in which the probability of jumping outside their base location and the agents’ randomness in the random walk are intermediate, in Fig. 4b. Finally, we investigate the case in which agents tend to spend most of their time outside their base location (large *p* and *α*), in Fig. 4c.

As expected from the formulation of the model, we determine that agents with larger radii of interaction tend to have larger average degrees. Also, agents with larger radii of interaction are more likely to contact agents outside of their base location, thereby leading to lower clustering coefficients $\mathcal {C}$ (Saramäki et al. 2007), which is a measure of the agents’ tendency to form clusters.^3^ The results of our simulations are reported in Figs. 4d–f. During the evolution of an epidemic process, agents with large radii might act as “superspreaders (Lloyd-Smith et al. 2005),” which are known to have a key role on the disease spreading, by creating many connections and infecting agents from different locations.

Less expected are the relationships between agents’ radii of interaction and their base location, and between agents’ clustering coefficients and their radii of interaction. Among the agents with a small radius of interaction, the agents that are assigned to central locations have a larger average degree than those that are assigned to peripheral locations. This result is independent of the time spent outside their base location (that is, independent of *p* and *α*). Interestingly, the same argument does not apply when agents have a large radius of interaction. In this situation, agents assigned to peripheral locations may have a larger degree than agents assigned to central locations, because their high radius of interaction allows a multitude of interactions, independent of the position of their base location. In addition, agents assigned to central locations have a lower clustering coefficient than agents assigned to peripheral locations. This is because the former group interacts with more agents and creates less tight clusters than the latter group that is assigned to peripheral locations.

Further, we comment that time spent outside the base location (regulated by *p* and *α*) is inversely proportional to the dispersion of the agents’ degree. In fact, the largest dispersion in the agents’ degree is registered when the probability of jumping outside the base location and the agent’s randomness are small, in Fig. 4a. Dispersion in the agents’ degree decreases as the probability of jumping outside the base location and the agent’s randomness increase, in Fig. 4b and in Fig. 4c. A possible explanation for this phenomenon can be based on the following argument. The more the agents spend time inside their base location, the more they remain isolated from other agents in the system. On the contrary, agents’ isolation is reduced when they spend more time outside their base location: they are able to interact with all the agents in the system, and, as a consequence, the dispersion in their degree decreases.

## Epidemic processes

Here, we investigate the spreading of epidemics over spatially-distributed populations that behave according to the presented agent-based model. Even though the complexity of the mobility mechanism and the presence of a geographical structure hinders the general mathematical treatment of the epidemics, some mathematical insight can be obtained by studying a simplified, one-dimensional version of the model.

We start by presenting the one-dimensional simplification, discussing our main analytical results and validating them against numerical simulations. Specifically, we focus on the impact of three salient model characteristics on epidemic processes. Namely, i) the random exploration of the space governed by the parameter *α*, ii) the probability of jumping outside the base location *p*, and iii) the presence of multiple locations. Interestingly, when multiple locations are present, the time spent inside the base location does not play an important role in the evolution of the contagion process.

Then, we consider the two-dimensional agent-based model and explore the effect of the same three salient model characteristics. We determine that results are qualitatively equivalent to those obtained in the one-dimensional case. We continue our numerical campaign on the two-dimensional agent-based model by studying whether the disease spreading is influenced by the presence of agents with larger radii in specific regions of the core-periphery structure. To this end, we study the presence of agents with greater radius of interaction in either the more central or more peripheral locations, thereby discovering that central locations are important for sustaining the overall diffusion. Finally, we analyze the outcome of vaccination strategies, finding that the highest beneficial effect for the entire population is registered when the vaccination of agents in central locations is prioritized.

We consider an infectious disease with the possibility of re-infection (SIS model) or immunization (SIR model), after the contraction of the infection. In the SIS model, agents can be either susceptible to the disease or infected (Keeling and Rohani 2011). Two mechanisms characterize the epidemic dynamics: infection propagation and recovery process. The former occurs when an infected agent contacts a susceptible one, who may become infected with a probability *λ*, independently of the others. The latter consists of the spontaneous transition from the infected state to the susceptible one and occurs with probability *μ* at each unit time, independently of the others. In the SIR model, instead, individuals who recover cannot be infected again and transition from the infected state to a removed state with probability *μ* per unit time (Keeling and Rohani 2011).

In the SIS model, we examine the endemic prevalence (that is, the number of active cases in the long-term), which has typically two possible outcomes: either it quickly dies out and tends to zero, or it fluctuates around a quantity greater than zero for a nonnegligible amount of time, denoted by *i*^∗^. For the SIR model, instead, the fraction of infected individuals in the system always goes to zero in the long-run. However, the total fraction of individuals who have been infected may vary, depending on the model parameters. The SIR epidemic size, denoted as *r*^*∞*^, is defined as the fraction of recovered individuals at the end of the epidemic process.

### One-dimensional model on a lattice

Here, we propose a one-dimensional model that provides some analytical intuitions on the influence that the randomness *α*, the probability of jumping outside the base location *p*, and the presence of a core-periphery structure have in the evolution of SIS and SIR epidemic processes. This model simplifies the two-dimensional case study by constraining agents to move in a discrete, infinitely long, one-dimensional lattice with periodic boundary conditions (that is, a ring). The *L* locations occupy consecutive positions on the lattice (labeled from 1 to *L*), and a fixed number of *n*=*N*/*L* agents belong to each one, as their base location.

**Fig. 5:**
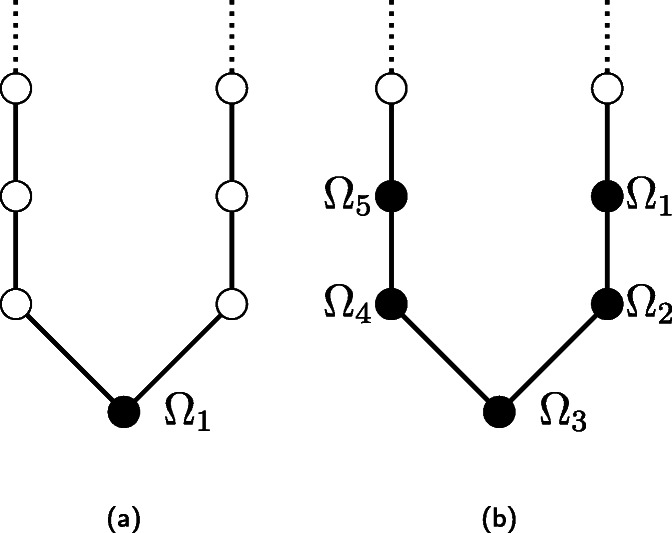
Schematic of the one-dimensional version of the agent-based model. **a** Scenario where only one base location is present (in black). **b** Scenario where multiple base locations are present

To generate a contact, agents should occupy the same position along the lattice. Agents belong to a unique base location in the lattice, which they may leave with probability *p*. We use a geometric distribution (Chung and Zhong 2001) to describe the agents’ law of motion, that is, the probability of jumping at a distance *d* from the base location is equal to

10 $$ P_{{jump}}(d) =\left(1-c\right)^{d-1}c\,,   $$

where *c*∈(0,1) is a constant parameter that governs the decay rate, similar to Eq. (6). Once outside their base location, agents move toward their base location by making one step toward it, similar to the two-dimensional model with *α*=0. A schematic representation of the one-dimensional model is provided in Fig. 5.

We remark that this one-dimensional model maintains some key features of the original two-dimensional agent-based model, that is: i) the presence of closely-spaced base locations, ii) a stochastic mechanism that governs the probability of jumping outside the base location, and iii) a gravity law that biases the agents to jump close to the base location according to an exponential distribution. A key feature that is not captured by this simplified model is the heterogeneity in the locations’ density and agents’ radii of interaction, which are numerically investigated in the two-dimensional model.

We start our analysis by reporting the probability that a generic agent $i\in \mathcal V$ is inside location *ℓ*, which is explicitly derived in Appendix C,

11 $$ q_{\ell} = \frac{c}{L(c+p)}+\frac1L \sum\limits_{x = 1}^{L-\ell} \frac{cp \left(1-c\right)^{x-1}}{2(c+p)} +\frac1L\sum\limits_{x = 1}^{\ell-1} \frac{cp \left(1-c\right)^{x-1}}{2(c+p)}\,.   $$

Similarly, the probability that a generic agent is in a position that is not occupied by any location and at a distance *d* from the closest location is computed in Appendix C as

12 $$ q_{\text{out}, d} = \frac{1}{L} \sum\limits_{x = d}^{L+d-1} \frac{cp \left(1-c\right)^{x-1}}{2(c+p)}\,,   $$

where we assume that the closest location is *ℓ*=1. By a simple change of variables, we can write an equivalent expression when the closest location is *ℓ*=*L*.

In the SIR and SIS processes, the disease propagates from infected agents to susceptible ones occupying the same position of the one-dimensional lattice. We define as *s*(*t*), *i*(*t*), and (for the SIR model only) *r*(*t*) the fractions of susceptible, infected, and recovered agents at time *t*, respectively. For large-scale systems, we can compute the fraction of susceptible, infected, and recovered agents along the lattice by using the law of large numbers (Chung and Zhong 2001). In the thermodynamic limit of large systems *N*→*∞*, the fraction of susceptible, infected, and recovered agents inside location *ℓ* is *s*_*ℓ*_(*t*)=*q*_*ℓ*_*s*(*t*), *i*_*ℓ*_(*t*)=*q*_*ℓ*_*i*(*t*), and *r*_*ℓ*_(*t*)=*q*_*ℓ*_*r*(*t*), respectively. Similarly, the fraction of susceptible, infected, and recovered agents at a distance *d* from the closest location is *s*_*out*,*d*_(*t*)=*q*_*out*,*d*_*s*(*t*), *i*_out,*d*_(*t*)=*q*_out,*d*_*i*(*t*), and *r*_*out*,*d*_(*t*)=*q*_*out*,*d*_*r*(*t*), respectively.

In the thermodynamic limit of large systems *N*→*∞*, the evolution of the fraction of infected agents at time *t*+1 is determined by the following equation:

13 $$ \begin{aligned} i(t+1) &=&i(t) - \mu i(t) + \sum\limits_{\ell = 1}^{L} s_{\ell}(t) \Lambda_{\ell}(t) + 2 \sum\limits_{d=1}^{\infty} s_{\text{out},d}(t) \Lambda_{\text{out},d}(t)\\ &=&i(t) - \mu i(t) + \sum\limits_{\ell = 1}^{L} q_{\ell} s(t) \Lambda_{\ell}(t) + 2 \sum\limits_{d=1}^{\infty} q_{\text{out},d}s(t) \Lambda_{\text{out},d}(t)\,, \end{aligned}   $$

where *Λ*_*ℓ*_(*t*) is the contagion probability of an agent inside its base location *ℓ* at time *t*, that is

14 $$ \Lambda_{\ell}(t) = 1- \left(1-\lambda i_{\ell}(t)\right)^{q_{\ell} N} = 1- \left(1-\lambda q_{\ell}i(t)\right)^{q_{\ell} N}\,,   $$

and *Λ*_out,*d*_(*t*) is the contagion probability of an agent at distance *d* from the closest location at time *t*, that is,

15 $$ \Lambda_{\text{out},d}(t) = 1- \left(1-\lambda i_{\text{out},d}(t)\right)^{q_{\text{out},d}N}= 1- \left(1-\lambda q_{\text{out},d}i(t)\right)^{q_{\text{out},d}N}\,.   $$

The derivation of these expressions is reported in Appendix C. The evolution of the fraction of infected agents in Eq. (13) depends on four terms: i) the fraction of infected at time *t*, ii) the fraction of newly recovered, iii) the fraction of newly infected in any location, and iv) the fraction of newly infected outside all the locations.

The evolution of the SIS model is fully determined by Eq. (13), since *s*(*t*)=1−*i*(*t*). For the SIR model, instead, Eq. (13) should be coupled with the following equation, which describes the evolution of the fraction of recovered agents,

16 $$ r(t+1) =r(t) + \mu i(t)\,,   $$

and with the conservation constraint *s*(*t*)=1−*r*(*t*)−*i*(*t*). The evolution of the fraction of recovered agents only depends on the fraction of recovered at time *t* and the fraction of newly recovered.

In order to gain qualitative insight into the behavior of the SIS and SIR epidemic processes described by Eqs. (13) and (16), we compute the epidemic threshold of both processes by studying the stability of the disease-free equilibrium in Eq. (13). We linearize Eq. (13) and expand the expressions for the contagion probabilities in Eqs. (14) and (15) about the disease-free equilibrium *i*^∗^=0, obtaining

17 $$ i(t+1) = i(t) - \mu i(t) + \sum\limits_{\ell = 1}^{L}\lambda q_{\ell}^{3}N i(t) + 2 \sum\limits_{d=1}^{\infty} \lambda q_{\text{out}, d}^{3}N i(t)\,.   $$

The epidemic threshold is computed by imposing *i*(*t*+1)≤*i*(*t*) in Eq. (17), obtaining

18 $$ \begin{aligned} \frac{\lambda}{\mu} \leq {\frac{1}{N\left(\sum\limits_{\ell = 1}^{L} q_{\ell}^{3} + 2 \sum\limits_{d=1}^{\infty} q_{\text{out}, d}^{3}\right)}}\,.  \end{aligned}  $$

In the case of one location, *L*=1, the threshold in Eq. (18) reduces to

19 $$ \frac{\lambda}{\mu} \leq \frac{1}{ N\left(q_{1}^{3}+ 2 \sum\limits_{d=1}^{\infty} q_{\text{out},d}^{3}\right)} =\frac{4 \left(c + p \right)^{3} \left(3 - 2c + c^{2} \right)}{N c^{2} \left(2 c \left(3 - 2c + c^{2} \right) + p^{3} \right)}\,,   $$

where the last equality is obtained by substituting the explicit terms for *q*_1_ and *q*_out,*d*_ from Eqs. (11) and (12), respectively, and computing the sum of the obtained series. From inspection of Eq. (19), we observe that increasing the probability of jumping outside the location, *p*, contributes to increasing the epidemic threshold and thus lowers the endemic prevalence and epidemic size.

When many locations are present, that is, *L*→*∞*, the second term at the denominator yields a marginal contribution to the epidemic threshold in Eq. (19), so that,

20 $$ \frac{\lambda}{\mu} \approx \frac{1}{ N\sum\limits_{\ell = 1}^{\infty} q_{\ell}^{3}}\,.   $$

We observe that the epidemic threshold is now independent from any choice of the probability of jumping outside the location, *p*.

**Fig. 6:**
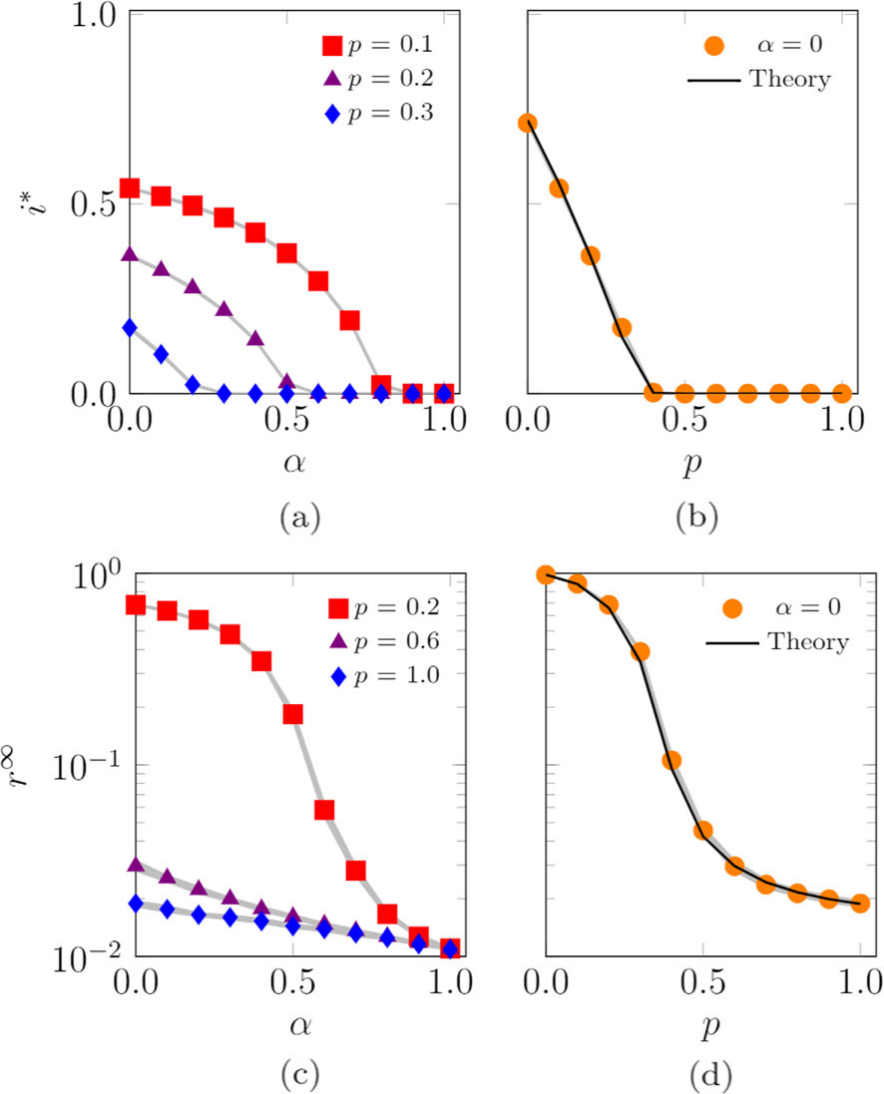
Influence of the agents’ randomness, *α*, and probability of jumping outside the location, *p*, on the SIS endemic prevalence, **a**-**b**, and SIR epidemic size, **c**-**d**. Theoretical values of the SIS endemic prevalence, **b**, and SIR epidemic size, **d**, are computed by evaluating the steady state in Eqs. (13) and (16), respectively. Curves represent the median of 100 independent simulations; 95% confidence bands are displayed in gray. Agents are initially inside their base location and the infection starts after 100 steps to allow the agents to reach a steady-state configuration. The fraction of randomly chosen initial infected agents is 0.01. Other parameter values are *L*=1, *N*=10,000, *D*=100,000, *r*=0.0004, *c*=0.3333, *λ*=0.1, and *μ*=0.1

**Fig. 7:**
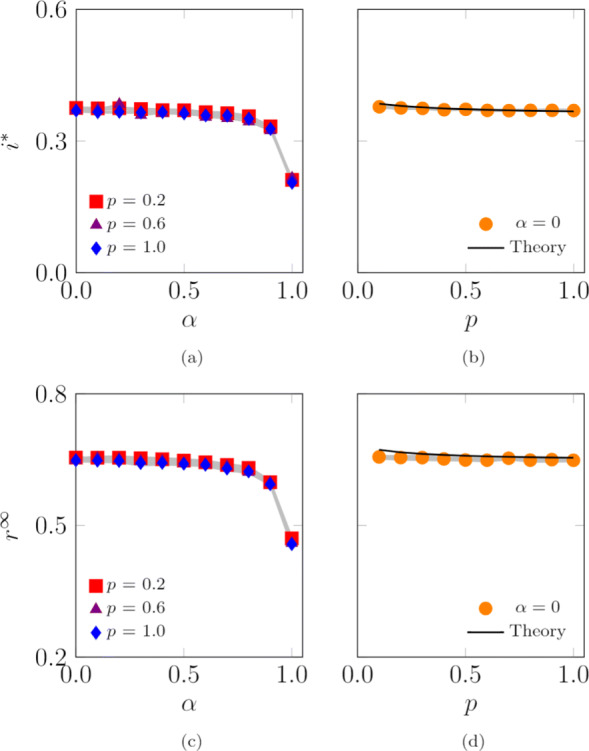
Influence of the agents’ randomness, *α*, and probability of jumping outside the location, *p*, on the SIS endemic prevalence, **a**-**b**, and SIR epidemic size, **c**-**d**. Theoretical values of the SIS endemic prevalence, **b**, and SIR epidemic size, **d**, are computed by evaluating the steady state in Eqs. (13) and (16), respectively. Curves represent the median of 100 independent simulations; 95% confidence bands are displayed in gray. Agents are initially inside their base location and the infection starts after 100 steps to allow the agents to reach a steady-state configuration. The fraction of randomly chosen initial infected is 0.01. Other parameter values are *L*=100, *N*=10,000, *D*=100,000, *r*=0.01, *c*=0.3333, *λ*=0.05, and *μ*=0.03

We conclude the analysis of the one-dimensional model by numerically studying the effect of the agents’ randomness *α* and of the probability of jumping outside the location *p* on the SIS endemic prevalence and the SIR epidemic size. These numerical simulations extend our analytical predictions, which are limited to the case *α*=0. We consider two scenarios, one with *L*=1 locations, presented in Fig. 6, and the other with *L*=100 locations, illustrated in Fig. 7. Our simulations suggest that increasing the agents’ randomness *α* hinders the diffusion of both SIS and SIR epidemic processes. When only one location is present, increasing the probability of jumping outside the location (that is, shortening the time spent inside the base location) hinders both SIS and SIR epidemic processes. Interestingly, when multiple locations are present, increasing *p* does not impact the evolution of the epidemic processes. Our numerics for *α*=0 in Figs. 6b,d and 7b,d indicate the potential use of the analytical expressions in Eqs. (13) and (16) for systems of finite size, with *N*=10,000 agents.

### Two-dimensional agent-based model

#### Impact of key parameters

We consider the two-dimensional agent-based model and numerically study the influence of the randomness *α*, the probability of jumping outside the base location *p*, and the presence of a core-periphery structure on the evolution of SIS and SIR epidemic processes. We start our analysis by exploring the case of a space containing one location, that is, *L*=1, which is the base for all the agents. Agents can be either inside or outside their base location. Their motion is constrained by the boundary of the location when they are inside it, while it is governed by the parameters *α* or *p* when they are outside their base location.

**Fig. 8:**
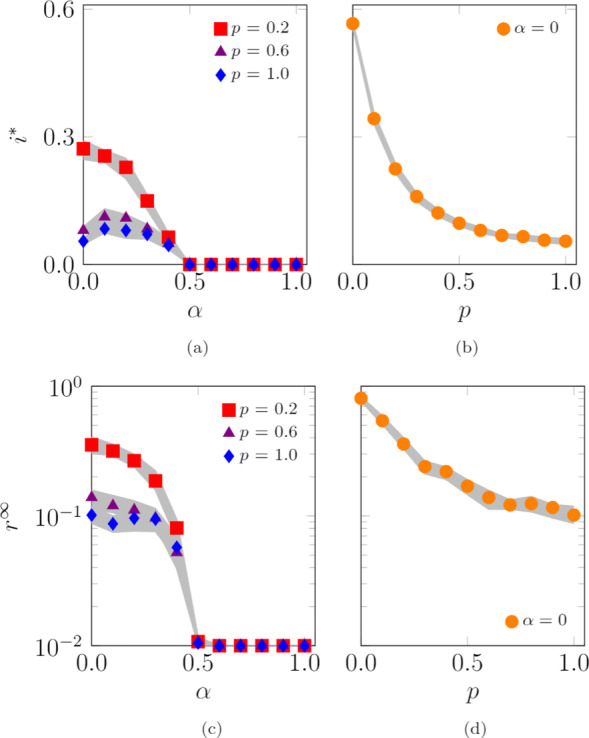
Influence of the agents’ randomness, *α*, and the probability of jumping outside the location, *p*, on the endemic prevalence of the SIS model, **a**–**b**, and the epidemic size of the SIR model, **c**–**d**. Curves represent the median of 100 independent simulations; 95% confidence bands are displayed in gray. Agents are initially inside their base location and the infection starts after 100 steps to allow the agents to reach a steady-state configuration. The fraction of randomly chosen initial infected is 0.01. Other parameter values are *L*=1, *N*=10,000, *D*=10^9^, *Σ*_min_=100, *Σ*_max_=10,000, *σ*_min_=1, *σ*_max_=1,000, *v*=500, *c*=4·10^−4^, *ω*=2.4, *γ*=2.1, *λ*=0.15, and *μ*=0.1

Our results reveal that increasing either *α* or *p* reduces the impact of the epidemic disease, both in the case of possible reinfection (SIS), as shown in Fig. 8a, and in the case of immunization after recovery (SIR), as illustrated in Fig. 8b. Specifically, in the SIS process, the endemic prevalence, *i*^∗^, is high when *α* and *p* are low because agents spend more time inside the location, which is the densest region of the entire space, thus favoring interactions between agents. On the contrary, when agents spend more time outside the location (by increasing either *α* or *p*^4^), they explore a less dense region of the space and interactions become more sporadic. As a result, the likelihood that the disease spreads is lower. From our numerical simulations, we observe that there is a threshold for *α* (for *α* close to $\bar \alpha =$ 0.5), beyond which the disease spreading is halted. Simulations with different values of the parameters show a similar behavior, with varying values of the threshold $\overline \alpha $. Hence, in the SIS dynamics, the disease is not able to spread and the endemic prevalence tends to zero, as shown in Fig. 8a; a similar behavior is observed for the SIR process. Similar results are obtained for the one-dimensional lattice, as illustrated in Fig. 6.

**Fig. 9:**
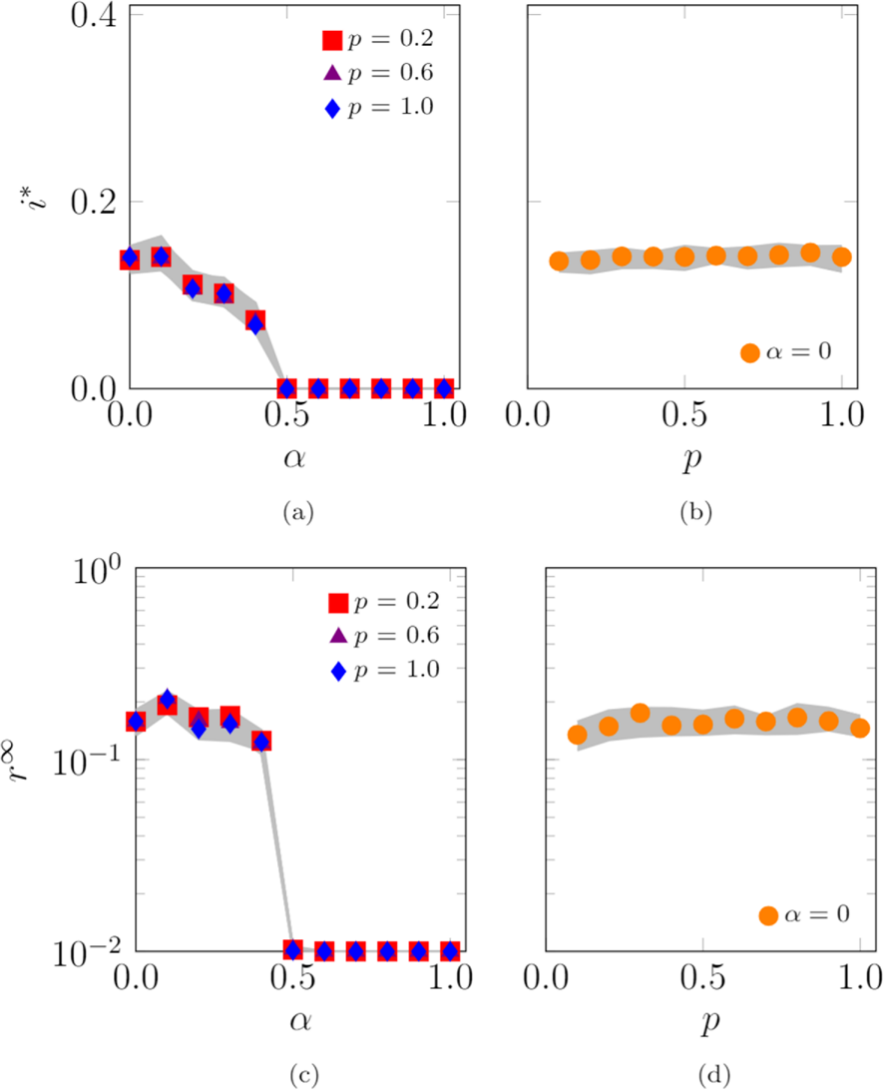
Influence of the agents’ randomness, *α*, and the probability of jumping outside the location, *p*, on the endemic prevalence (SIS model), **a**–**b**, and the epidemic size (SIR model), **c**–**d**. Curves represent the median of 100 independent simulations; 95% confidence bands are displayed in gray. Agents are initially inside their base location and the infection starts after 100 steps to allow the agents to reach a steady-state configuration. The fraction of randomly chosen initial infected is 0.01. Other parameter values are *L*=100, *N*=10,000, *D*=10^9^, *Σ*_min_=100, *Σ*_max_=10,000, *σ*_min_=1, *σ*_max_=1,000, *v*=500, *c*=4·10^−4^, *ω*=2.4, *γ*=2.1, *λ*=0.15, and *μ*=0.1

Next, we consider the case in which multiple locations are present, forming a core-periphery structure, as described in Appendix A and illustrated in Fig. 1b. Agents that exit their base location are likely to jump inside another location and interact with other agents occupying a different portion of the urban environment. We investigate a scenario with *L*=100 locations, as illustrated in Fig. 9. Our numerical results suggest that increasing the agent’s randomness, *α*, still reduces the endemic prevalence (in the SIS model) and the epidemic size (in the SIR model), *i*^∗^ and *r*^*∞*^, similar to the case of a single location. Numerical results in Fig. 9a and c, however, seem to display a nonmonotonic behavior of the fraction of population affected by the disease, whereby small values of *α* may favor the epidemic outbreak instead of hindering its inception. We record the existence of a threshold for *α* (in our simulations, this is close to 0.5) at which a sharp transitions takes place for both the endemic prevalence (in the SIS model) and the epidemic size (in the SIR model). According to Eq. (3), by increasing *α*, agents’ randomness is increased and, as a consequence, agents tend to explore a larger portion of the urban environment and to occupy peripheral locations with a lower density of agents. Hence, they become are less likely to interact with each other and support disease spreading.

Surprisingly, we observe that the probability of jumping outside the base location, *p*, seems to have a negligible effect on the outcome of the SIS and SIR disease processes, similar to predictions from the one-dimensional simplified version of the model in Eq. (20) and Fig. 7. A reason for this phenomenon may be found in the following intuition. The core-periphery structure analyzed in our work, illustrated in Fig. 1, allows two contrasting effect to simultaneously occurs. On the one hand, agents moving outside the central areas are likely to end in peripheral ones, decreasing the agents’ density in the central regions and increasing the density in the peripheral ones. On the other hand, agents moving outside the peripheral areas are likely to end in the central ones, thereby increasing the density in the central regions and decreasing the density in the peripheral ones. Overall, these two opposite effects tend to balance each other.

#### Impact of the correlation between agents’ radius and locations’ density

**Fig. 10:**
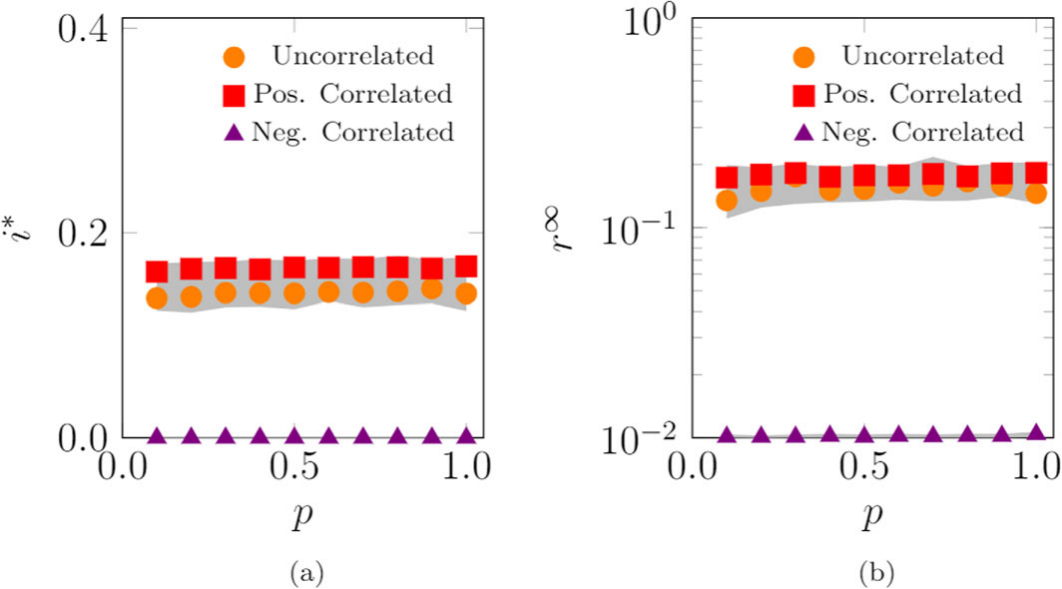
Impact of different ways of assigning agents to their locations on the endemic prevalence (SIS model), **a**, and the epidemic size (SIR model), **b**. The “Uncorrelated” case represents a random assignment. In the “Pos. Correlated” case, agents with larger radii are assigned to the denser (central) locations, while, in the “Neg. Correlated” case, agents with larger radii belong to the less dense (peripheral) locations. Curves represent the median of 100 independent simulations; 95% confidence bands are displayed in gray. Agents are initially inside their base location and the infection starts after 100 steps to allow the agents to reach a steady-state configuration. The fraction of randomly chosen initial infected is 0.01. Other parameter values are *L*=100, *N*=10,000, *D*=10^9^, *Σ*_min_=100, *Σ*_max_=10,000, *σ*_min_=1, *σ*_max_=1,000, *v*=500, *c*=4·10^−4^, *ω*=2.4, *γ*=2.1, *λ*=0.15, and *μ*=0.1

Here, we study the impact of the correlation between the radius of interaction of agent *i*, *σ*_*i*_, and the density of its base location, *ρ*_*β*(*i*)_. We compare the uncorrelated case (analyzed earlier in Fig. 9a and c), where agents are randomly assigned to locations, with the cases of either positive or negative correlation. In the case of positive correlation, agents with larger radius are assigned to denser (and central) locations. In the case of negative correlation, agents with larger radius belong to the less dense (and peripheral) locations. We consider a scenario with *L*=100 locations, whose results are illustrated in Fig. 10.

Both the endemic prevalence, *i*^∗^, and the epidemic size, *r*^*∞*^, are marginally affected by a positive correlation, while they strongly diminish if the radii and density of locations are negatively correlated, as shown in Fig. 10a and b, respectively. In both the positive-correlated and uncorrelated cases, agents with larger radii occupy the central locations, thereby sustaining the diffusion of the disease. On the other hand, if agents with large radii are relegated to peripheral and sparser areas, it would be more difficult for them to create connections and fuel the diffusion process.

#### Vaccination strategies

Finally, we examine the effect of different vaccination strategies applied to our population. Specifically, we consider a purely randomized strategy and two targeted vaccination policies. In the three strategies, we assume that a fraction of the population is vaccinated and is thus immune to the disease. In the “Random” vaccination mechanism, we vaccinate a fraction of the population, sampled uniformly at random. In the “Center” targeted mechanism, we select such a fraction starting from the agents assigned to the most central locations. In the “Peripheral” targeted mechanism, we choose such a fraction starting from the agents assigned to the most peripheral locations.

**Fig. 11:**
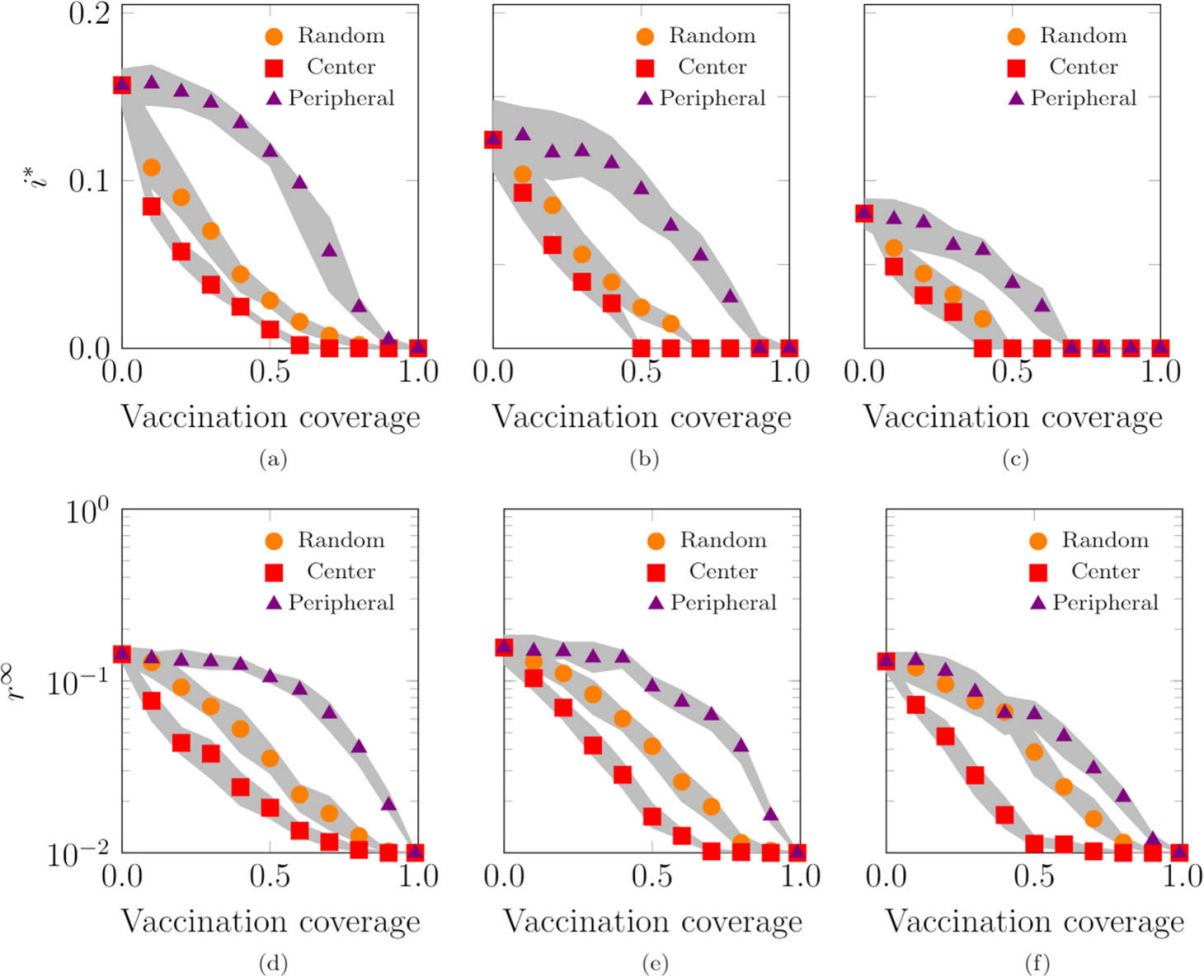
Effect of different vaccination strategies on the endemic prevalence (SIS model), **a**-**b**-**c**, and epidemic size (SIR model), **d**-**e**-**f**. The vaccination coverage represents the fraction of immune agents prior to the disease onset. In “Random”, we select the fraction of agents to vaccinate at random; in “Center”, we vaccinate first the agents that are assigned to central base locations, while in “Peripheral”, we prioritize vaccination for agents that belongs to the peripheral agents. We set: **a**-**d** *p*=0.1 and *α*=0.0, **b**-**e** *p*=0.4 and *α*=0.2, and **c**-**f** *p*=0.8 and *α*=0.4. Curves represent the median of 100 independent simulations; 95% confidence bands are displayed in gray. Agents are initially inside their base location and the infection starts after 100 steps to allow the agents to reach a steady-state configuration. The fraction of randomly chosen initial infected is 0.01. Other parameter values are *L*=100, *N*=10,000, *D*=10^9^, *Σ*_min_=100, *Σ*_max_=10,000, *σ*_min_=1, *σ*_max_=1,000, *v*=500, *c*=4·10^−4^, *ω*=2.4, *γ*=2.1, *λ*=0.15, and *μ*=0.1

From Fig. 11, we observe that prioritizing the vaccination of agents assigned to the most central locations has the most beneficial effect for the prevention of the diffusion of the epidemic disease, while the worst strategy targets vaccination to peripheral areas. As detailed in Fig. 4, agents assigned to more central base locations tend to have a larger expected degree than agents assigned to more peripheral locations, thereby potentially acting as “superspreaders” (Lloyd-Smith et al. 2005). Also, agents whose base locations are in the center can easily reach all portions of the environment, thereby contacting the majority of the agents. By focusing the vaccination on central areas, the contacts generated by these agents do not contribute to the spread, thereby significantly reducing the contagion.

## Discussion and conclusion

In this paper, we studied a class of agent-based models (Frasca et al. 2006), in which agents move in a two-dimensional space and interact according to proximity criteria. We extended such class of models by encapsulating a core-periphery structure, typical of urban environments (Makse et al. 1998; De Nadai et al. 2016), where central areas are more densely populated than peripheral ones. Our urban-like environment is partitioned in several closely spaced locations, each of them representing a restricted portion of the space. When agents are inside their base location, they take a random position within the base location at every time-step. When outside, they tend to move back to their base location by following a biased random walk.

The contribution of the study is fourfold. First, we analytically and numerically studied the temporal network formation mechanism, demonstrating that heterogeneously distributed radii of interaction in the population generate heterogeneity in the degree distribution of the temporal network of contacts, similar to what is observed in many real-world systems (Barabási and Albert 1999; Albert et al. 1999; Barrat et al. 2004). The role of the interaction radius is also evident in the study of the clustering coefficient, whereby we found that agents’ with larger degree have a lower clustering coefficient.

Second, we investigated the role of the urban-like environment on the spread of epidemic outbreaks. Specifically, we considered epidemic prevalence in the susceptible–infected–susceptible (SIS) model and epidemic size in the susceptible–infected–recovered (SIR) model. We found that both these quantities, which measure the fraction of the system that is affected by the disease, are lowered by increasing the randomness of the agents’ law of motion. In fact, increasing agents’ randomness improves the chance that agents randomly explore peripheral urban areas, where less agents are present and less contacts are thus created. A lower number of interactions hinders the contagion process. Interestingly, we discovered that the endemic prevalence and epidemic size have nontrivial relationships with the probability of jumping outside the base location. When the entire urban environment is modeled as a unique location, larger probabilities of jumping outside hinder the epidemic diffusion. In fact, inside the location the density of agents is higher than outside it. As a consequence, interactions between agents are rare, slowing down the disease spread. Instead, when multiple locations are arranged in a core-periphery structure, our numerical results suggest that epidemic prevalence and size are independent of the probability of jumping outside the base location. A possible explanation for this phenomenon might be that, when agents in central locations jump outside them, they are likely to end in peripheral locations, diminishing the fraction of agents in central areas. This event is compensated by agents from peripheral locations that jump in central ones. Our numerical results are in agreement with the theoretical findings in the simplified, one-dimensional, version of our agent-based model.

Third, we found that central locations play a key role on the diffusion of epidemic diseases. In particular, we studied the influence of the correlation between agents’ radius and locations’ density. When these quantities are negatively correlated, agents with larger radius belong to less dense (peripheral) locations, while when positively correlated, agents with larger radius belong to denser (central) locations. The endemic prevalence (in the SIS model) and the epidemic size (in the SIR model) are only marginally favored by the presence of many agents with large radius in the more central locations (positive correlation), while the diffusion of the epidemic is hindered if central locations are mostly assigned to agents with small radius of interaction (negative correlation).

Finally, we studied the effect of targeted vaccination strategies. We found that the vaccination of agents that belong to central locations is the most beneficial approach for the entire population, leading to the smallest epidemic prevalence. Our analysis corroborates our previous observation that central (and more dense) locations are crucial in the diffusion of disease processes. We emphasize that the proposed vaccination strategy can be implemented with information about the system at the mesoscopic level of locations, that is, without any information on the specific properties of single individuals (for instance, their radius of interaction). With information at the individual level, the proposed policy may be improved by combining knowledge about locations and radii of interaction prioritizing vaccination of central agents with large radius of interaction, which acts as “superspreaders.”

A main limitation of our work resides in the assumption that each agent belongs to a unique location, while the remaining urban area, occupied by other locations, is only seldom explored. A more realistic approach could consider agents that may be assigned to multiple locations. Our theoretical study of the one-dimensional case provides insight into some aspects of epidemic processes in urban environments. However, a general mathematical theory is missing. We believe that our preliminary results constitute a starting point for performing a more general theoretical analysis of the two-dimensional model. Furthermore, variations of the proposed model can be easily generated. For instance, the gravity law in our model could be replaced by other laws, such as, the well-established radiation law (Simini et al. 2012) or the one recently proposed in (Schläpfer et al. 2020).

Overall, our work determines that central urban areas are critical in the diffusion of epidemic diseases within a city, being the crossroad of most of the urban population, and thus should be carefully included into mathematical models of epidemic outbreaks. By vaccinating individuals in central urban areas, we can halt the overall contagion better than randomly distributing limited vaccination supplies. Our proposed vaccination strategy may offer practitioners and epidemiologists general guidelines for emergency situations, complementing other strategies (Braha and Bar-Yam 2006; Génois et al. 2015) toward effective containment measures and herd immunity (Fine 1993) within urban environments.

## Appendix A: Algorithm to generate a core-periphery structure

From a practical point of view, packing all convex regions *L*_*ℓ*_ (locations) in the square space *D*×*D* is a nondeterministic polynomial-time hardness (NP-hard) problem (Martello and Vigo 1998; Demaine et al. 2010), often requiring to find approximate methods (Formann and Wagner 1991; Szabó et al. 2007; Castillo et al. 2008). In our study, we aim at reproducing the core-periphery structure present in real urban areas, as shown in Fig. 1a, while minimizing the space between locations. Agents that exit from their base location occupy nearby locations, thereby interacting with agents that are assigned to different regions of the urban area. We developed a heuristic algorithm that unfolds according to the following steps.

1. Place the center of the denser location, $\left (x_{1}^{c}, y_{1}^{c}\right)$, in the center of the square space, $\left (x_{1}^{c}, y_{1}^{c}\right) = \left ({D}/2, {D}/2 \right)$.
2. Initialize *ℓ*←1, *σ*_in_←0, and $\sigma _{\text {out}}\leftarrow \langle {{\sigma }\rangle } = {\sum \nolimits }_{\ell =1}^{L} {\sigma }_{\ell }/L$.
3. Create a circular crown centered in (*D*/2,*D*/2) with internal radius *σ*_in_ and external radius *σ*_out_.
4. Randomly place the center of location *ℓ*+1 in the crown and check for overlaps.
  i) If location *ℓ*+1 does not overlap with other locations, then the location is placed. Increase the index *ℓ* by 1, that is, *ℓ*←*ℓ*+1. If *ℓ*=*L*, then terminate the algorithm. Otherwise, resume it to step 4.
  ii) If an overlap occurs, then repeat the current assignment in 4. After a number of consecutive failed attempts (we set this limit to 100), stop the current iteration and move to step 5.
5. Set *σ*_in_←*σ*_out_ and *σ*_out_←*σ*_out_+〈*σ*〉, and resume the algorithm to step 3.

## Appendix B: Analysis of the temporal network of interactions

Here, we detail the analytical derivations of Eqs. (8) and (9). To this end, we analyze the formation of the temporal network of interactions in the two specific cases of a free space, without any location (*L*=0); and when the law of motion of the agents outside their base locations is deterministic (*α*=0) and the locations are uniformly distributed in the plane.

### B.1 Analysis of a free space without any location

We begin our analysis by considering the case of a free space, that is, *L*=0, where agents perform simple random walks with constant velocity equal to *v* in the plane. In this scenario, Eq. (3) should be intended without the component associated with the location *Φ*_*i*_(*t*) and with *α*=1.

According to Eq. (7), at time *t*, agent *i* creates undirected interactions with other agents if their Euclidean distance is less than or equal to the maximum of their radii of interaction. In practice, the expected number of interactions of agent *i*, E[*k*_*i*_], is equal to the sum of two contributions: the expected number of interactions that are generated by agent *i* with agents that are in its radius of interaction, denoted as $\mathrm {E}\left [k^{+}_{i}\right ]$, and the expected number of interactions that are generated by other agents with *i*, denoted as $\mathrm {E}\left [k^{-}_{i}\right ]$.

When the system is in its steady state, the expected number of other agents within a distance *σ*_*i*_ from agent *i* is proportional to the ratio between the area of a circle of radius *σ*_*i*_ and the whole planar space. Hence, the expected number of interactions created by agent *i* is equal to

21 $$ \mathrm{E}\left[k^{+}_{i}\right]=\frac{\pi \sigma^{2}_{i}}{D^{2}}~(N-1)\,.   $$

Further, agent *i* can form undirected interactions with other agents if it is located within their radii of interaction. To avoid double counting and exclude connections that are already counted in Eq. (21), the radius of agent *j* should be greater than the one of agent *i*, which should be at a distance greater than *σ*_*i*_ but smaller than *σ*_*j*_. When the system reaches the steady state, the probability of such an event is $\pi \left (\sigma _{j}^{2}-\sigma _{i}^{2}\right)/D^{2}$. Let us introduce the set $\mathcal {C}_{i}$ of agents with radius of interaction greater than *σ*_*i*_ and let us define ${\left \langle \sigma ^{2}\right \rangle }_{i}=|\mathcal C_{i}|^{-1}{\sum \nolimits }_{j\in \mathcal C_{i}}\sigma ^{2}_{j}$ as their average square radius. The expected number of connections formed by agent *i* with other agents beyond those included in Eq. (21) is

22 $$ \mathrm{E}\left[k_{i}^{-}\right]=\frac{1}{D^{2}}\pi \sum\limits_{j\in\mathcal C_{i}}~\left(\sigma_{j}^{2}-\sigma_{i}^{2}\right)=\frac{\pi}{D^{2}}|\mathcal C_{i}|{\left({\left\langle\sigma^{2}\right\rangle}_{i}-\sigma_{i}^{2}\right)}\,.   $$

By summing Eqs. (21) and (22), we conclude that the average number of agents that an agent interacts with in a unit time, termed its average degree *k*_*i*_, is equal to

23 $$ \mathrm{E} \left[k_{i} \right] =\mathrm{E} \left[k^{+}_{i} \right] +\mathrm{E} \left[k^{-}_{i} \right] = \frac{ \pi}{D^{2}} \left(\left(N - 1\right) \sigma^{2}_{i} + \lvert{\mathcal{C}_{i}\rvert} \left({\left\langle{\sigma^{2}}\right\rangle}_{i} - \sigma^{2}_{i} \right)\right)\,.   $$

The computation of the quantities $|\mathcal C_{i}|$ and 〈*σ*^2^〉_*i*_, can be performed in the limit of large systems *N*→*∞*, by means of the strong law of large numbers (Chung and Zhong 2001). We start by explicitly writing the probability density function *G*(*σ*) of the power law distribution of the radii of interaction with cutoffs *σ*∈[*σ*_min_,*σ*_max_], as

24 $$ G(\sigma)=\left\{\begin{array}{ll}\frac{\omega-1}{\sigma_{\text{min}}^{1-\omega}-\sigma_{\text{max}}^{1-\omega}}\sigma^{-\omega}&\text{ if }\sigma\in\left[\sigma_{\text{min}},\sigma_{\text{max}}\right]\,,\\0&\text{otherwise\,,}\end{array}\right.  $$

where *ω* is the exponent. From the expression of *G*(*σ*), we compute |*C*_*i*_| using the strong law of large numbers (Chung and Zhong 2001), which ensures that almost surely

25 $$ \underset{N\to\infty}{\lim}\frac{|\mathcal C_{i}|}{N}=\int_{\sigma_{i}}^{\sigma_{\text{max}}}G(\sigma)d\sigma = \frac{\sigma_{i}^{1-\omega}-\sigma_{\text{max}}^{1-\omega}}{\sigma_{\text{min}}^{1-\omega}-\sigma_{\text{max}}^{1-\omega}}\,.   $$

We define the conditional probability density function

26 $$ G_{i}(\sigma):=G\left(\sigma\,|\, \sigma\geq \sigma_{i}\right)=\frac{G(\sigma)}{P\left[\sigma>\sigma_{i}\right]}=\frac{G(\sigma)}{\int_{\sigma_{i}}^{\sigma_{\text{max}}}G(\sigma)d\sigma}=\frac{\omega-1}{\sigma_{i}^{1-\omega}-\sigma_{\text{max}}^{1-\omega}}\sigma^{-\omega}\,,   $$

where the first equality holds due to scale invariance of the power law distribution, and then explicit computation is performed using the expression of *G*(*σ*). Using again the strong law of large numbers (Chung and Zhong 2001) and Eq. (26), we compute 〈*σ*^2^〉_*i*_ as

27 $$ \underset{N\to \infty}{\lim}{\left\langle{\sigma^{2}}\right\rangle}_{i}=\int_{\sigma_{i}}^{\sigma_{\text{max}}}\sigma^{2}G_{i}(\sigma)d\sigma= \frac{(\omega-1)\left(\sigma_{\text{max}}^{3-\omega}-\sigma_{i}^{3-\omega}\right)}{(3-\omega)\left(\sigma_{i}^{1-\omega}-\sigma_{\text{max}}^{1-\omega}\right)}\,,   $$

almost surely.

Finally, by substituting Eqs. (25) and (27) in Eq. (23), the expected degree of agent *i* in the limit of large systems, *N*→*∞*, almost surely reads

28 $$ \mathrm{E} \left[k_{i} \right] = \frac{{N} \pi}{D^{2}} \left(\left(1-\frac{\sigma_{i}^{1-\omega}-\sigma_{\text{max}}^{1-\omega}}{\sigma_{\text{min}}^{1-\omega}-\sigma_{\text{max}}^{1-\omega}}\right)\sigma^{2}_{i}+ \frac{(\omega-1)\left(\sigma_{\text{max}}^{3-\omega}-\sigma_{i}^{3-\omega}\right)}{(3-\omega)\left(\sigma_{\text{min}}^{1-\omega}-\sigma_{\text{max}}^{1-\omega}\right)}\right)\,,  $$

neglecting the terms on smaller order in *N*.

### B.2 Analysis of multiple locations uniformly distributed in the space

Now, we consider the limit case in which agents move straight toward their base location, that is, *α*=0, and we assume that locations are uniformly distributed in the planar space.

We consider the generic agent *i* that belongs to location *Ω*_*β*(*i*)_. Since *A*_*β*(*i*)_≪*D*^2^, we use the approximation *D*→*∞*. The probability for this agent to be in its base location, *q*_in_, can be computed by introducing the following partition of the planar space,

29 $$ C^{(i)}_{h}:=\left\{(x,y)\in[0,D]^{2} :(h-1)v< \underset{\left(\xi,\eta\right)\in \Omega_{\beta(i)}}{\min}\sqrt{\left(x-\xi\right)^{2}+(y-\eta)^{2}}\leq hv\right\},  $$

for any $h\in \mathbb Z_{\geq 0}$. Note that $C^{(i)}_{h}$ is the region of the plane from which agent *i* reaches its base location *β*(*i*) in exactly *h* time-steps. Consequently, when *h*=0 agents are inside their base location, that is, $C^{(i)}_{0}=\Omega _{\beta (i)}$. Any point (*x*,*y*) of the *D*×*D* planar space can be mapped onto this partition through the projection $z^{(i)}:[0,D]\times [0,D]\longrightarrow \mathbb {Z}_{\geq 0}$, defined as

30 $$ z^{(i)}(x,y)=h\iff (x,y)\in C^{(i)}_{h}\,.   $$

Using the mapping *z*^(*i*)^, for each agent $i\in \mathcal V$, we define the stochastic process $z_{i}(t):\mathbb Z_{\geq 0}\longrightarrow \mathbb Z_{\geq 0}$ as *z*_*i*_(*t*):=*z*^(*i*)^(*x*_*i*_(*t*),*y*_*i*_(*t*)). Since *α*=0, when an agent is outside its base location, then its law of motion is purely deterministic and it moves in the direction of the location. Therefore, if *z*_*i*_(*t*)=*h*≠0, then, *z*_*i*_(*t*+1)=*h*−1. If *z*_*i*_(*t*)=0, the agent is inside its base location, from which it exits only through a jump, which is statistically characterized by Eq. (6). Hence, with probability 1−*p* the process *z*_*i*_(*t*) remains in state 0 at the following time-step. Else, if a jump occurs, the process *z*_*i*_ evolves to state *h* with probability equal to

31 $$ q_{h}=\int_{(h-1)v}^{hv}P_{\text{jump}}(x)\,dx=\int_{(h-1)v}^{hv}ce^{-cx}\,dx = e^{-cv(h-1)}-e^{-cvh}\,.   $$

**Fig. 12:**
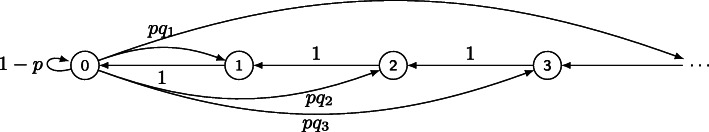
Transition graph of the Markov chain *z*_*i*_(*t*)

The transition probabilities of *z*_*i*_(*t*) depend only on the state *h* in which the process is and on the model parameters. The process *z*_*i*_(*t*) is a Markov chain, whose structure is illustrated in Fig. 12 and whose transition matrix is

32 $$ M =\left[\begin{array}{cccccc} 1-p&{pq}_{1}&{pq}_{2}&{pq}_{3}&\dots&\\ 1&0&0&0&\dots\\ 0&1&0&0&\dots\\ 0&0&1&0&\dots\\ \vdots&\vdots&\vdots&\ddots&\ddots\\ \end{array}\right]\,.  $$

We observe that, if *p*>0, then the Markov chain is ergodic and it converges to its unique stationary distribution *π*, which can be computed as the left eigenvector of *M* associated with the eigenvalue 1 (Levin et al. 2006). When the system has reached its steady state, the probability for each agent to be inside its base location, *q*_in_=*π*_0_, that is derived from the left eigenvalue equation for M in Eq. (32) (with unitary eigenvalue), that is,

33 $$ \left\{\begin{array}{lc}\pi_{0}= (1-p)\pi_{0}+\pi_{1},\\ \pi_{h}=\pi_{h+1}+{pq}_{h}\pi_{0}\,,\qquad \forall\,h\in\mathbb Z_{>0}\,. \end{array}\right.   $$

From Eq. (33), the expression of *q*_*h*_ in Eq. (31), and using that ${\sum \nolimits }_{h=0}^{\infty } \pi _{h}=1$, we derive

34 $$ q_{\text{in}} =\pi_{0} =\frac{e^{cv}-1}{(1+p)e^{cv}-1}\,.  $$

When the system reaches its stationary state, the number of agents in location *ℓ* is equal to the sum of two contributions. The first one consists of agents whose base location is *Ω*_*ℓ*_ and are in that location, that is, on average, *nq*_in_. The second one is due to agents whose base location is not *Ω*_*ℓ*_, but are in *Ω*_*ℓ*_. The second contribution is relatively small since locations are placed randomly in the entire space *D*×*D*, and we discard it when the system is large.

The steady-state density in location *ℓ* can be approximated by considering only the agents assigned to it. Hence, the expected number of connections of agent *i* within its base location is approximated by

35 $$ \mathrm{E} \left[k_{\text{in}, i} \right] \approx \frac{q_{\text{in}}}{\Sigma^{2}_{\ell}} \left(\left(n - 1\right)q_{\text{in}} \sigma^{2}_{i} + {\left\lvert{\mathcal{C}}_{i,\ell}\right\rvert} q_{\text{in}} \left({\left\langle{\sigma^{2}}\right\rangle}_{i,\ell} - \sigma^{2}_{i} \right)\right)\,,   $$

where $\mathcal C_{i,\ell }$ and 〈*σ*^2^〉_*i*,*ℓ*_ are the set of agents with radius greater than *σ*_*i*_ in location *ℓ* and their average square radius, respectively. Assuming the distribution of the radii of interaction to be independent of the agents’ base locations, then, $\left |\mathcal C_{i,\ell }\right |=\frac {n-1}{N}\mathcal C_{i}$ and 〈*σ*^2^〉_*i*,*ℓ*_=〈*σ*^2^〉_*i*_. Under this assumption, in the limit of large systems, *N*→*∞*, combining Eqs. (25) and (27) into Eq. (35), we obtain

36 $$ \mathrm{E} \left[k_{\text{in}, i} \right] \approx \frac{q^{2}_{\text{in}}}{\Sigma_{\ell}^{2}}~(n-1) \left(\sigma^{2}_{i} + \frac{\sigma_{i}^{1-\omega}-\sigma_{\text{max}}^{1-\omega}}{\sigma_{\text{min}}^{1-\omega}-\sigma_{\text{max}}^{1-\omega}} \left(\frac{(\omega-1)\left(\sigma_{\text{max}}^{3-\omega}-\sigma_{i}^{3-\omega}\right)}{(3-\omega)\left(\sigma_{i}^{1-\omega}-\sigma_{\text{max}}^{1-\omega}\right)}- \sigma^{2}_{i} \right)\right)\,.   $$

When a core-periphery structure is present, as in Fig. 1, locations are not uniformly distributed in space and often are close to each other. For instance, a central location *ℓ* is surrounded by other locations and interactions generated by agents whose base location is not *Ω*_*ℓ*_ cannot be neglected. This case is discussed in the main text by means of numerical simulations.

## Appendix C: Computation of the contagion probability in a one-dimensional lattice

Here, we compute the contagion probability of an agent inside its base location *ℓ* at time *t*, *Λ*_*ℓ*_(*t*), and the contagion probability of an agent at distance *d* from the closest location at time *t*, *Λ*_out,*d*_(*t*). We start our analysis by computing the probability that agents are in their base location, denoted by *ψ*_in_, or in a position at a distance *d* from it, *ψ*_*d*_, when the system is at steady state. For *p*>0, the system is ergodic and we can compute *ψ*_in_ and *ψ*_*d*_ at steady state (Levin et al. 2006). Similar to Appendix B, from the steady-state equation we derive the following recursive system of equations:

37 $$ \left\{\begin{array}{l}\psi_{\text{in}}= (1-p)\psi_{\text{in}}+2\psi_{1}\,,\\ \psi_{d}=\psi_{d+1}+\frac{p}{2} P_{\text{jump}}(d)\psi_{\text{in}}\,,\qquad \forall\,d\in\mathbb Z_{>0}\,,\end{array}\right.   $$

where the factor 2 is because there are two positions at a distance *d* from any location $\ell \in \mathcal {L}$, as in Fig. 5.

From Eq. (37), the expression of *P*_jump_(*d*) in Eq. (10), and using that $\psi _{\text {in}}+2 {\sum \nolimits }_{d=1}^{\infty } \psi _{d}=1$, we derive

38 $$ \psi_{\text{in}} = \frac{c}{c+p}\,,   $$

and

39 $$ \psi_{d} = \frac{cp \left(1-c\right)^{d-1}}{2(c+p)}\,.   $$

Given that each agent is randomly assigned to one of the *L* locations, the probability that a generic agent $i\in \mathcal V$ is inside location *ℓ* is equal to

40 $$ q_{\ell} = \frac{1}{L}\psi_{\text{in}} +\frac1L \sum\limits_{x = 1}^{L-\ell} \psi_{x} +\frac1L\sum\limits_{x = 1}^{\ell-1} \psi_{x}\,,   $$

where the first term refers to the probability that the agent is in its base location and its base location is *ℓ*, while the second and third terms correspond to the probability that the agent belongs to another base location and it occupies location *ℓ*. Similarly, we compute the probability that agents are in a position not occupied by any location and at a distance *d* from the closest location as

41 $$ q_{\text{out}, d} = \frac{1}{L} \sum\limits_{x = d}^{L+d-1} \psi_{x}\,,   $$

where we assume that the closest location is *ℓ*=1. Through a simple change of variables, we can write an equivalent expression when the closest location is *ℓ*=*L*. Substituting expressions in Eqs. (38) and (39) in Eqs. (40) and (41) yields the two expressions reported in the main text, that is, Eqs. (11) and (12).

We now compute the probability that an agent becomes infected at time *t*. We first consider the probability of not being infected. In location *ℓ*, such a probability is equal to 1−*λi*_*ℓ*_(*t*) for each contact. On average, an agent contacts *q*_*ℓ*_*N* other agents, the probability of not being infected in location *ℓ* is equal to $\overline {\Lambda }_{\ell }(t) = \left (1-\lambda i_{\ell }(t)\right)^{q_{\ell } N}$. Similarly, the probability of not being infected at a distance *d* from the closest location is equal to $\overline {\Lambda }_{\text {out},d} = \left (1-\lambda i_{\text {out},d}(t)\right)^{q_{\text {out},d} N}$. Thus, the contagion probability of an agent inside its base location *ℓ* is the complement of $\overline {\Lambda }_{\ell }(t)$, that is,

42 $$ \Lambda_{\ell}(t) = 1- \left(1-\lambda i_{\ell}(t)\right)^{q_{\ell} N} = 1- \left(1-\lambda q_{\ell}i(t)\right)^{q_{\ell} N}\,.  $$

Likewise, the contagion probability when the agent is at a distance *d* from the closest location is the complement of $\overline {\Lambda }_{\text {out},d}(t)$, that is,

43 $$ \Lambda_{\text{out},d}(t) = 1- \left(1-\lambda i_{\text{out},d}(t)\right)^{q_{\text{out},d}N}= 1- \left(1-\lambda q_{\text{out},d}i(t)\right)^{q_{\text{out},d}N}\,.  $$

## Data Availability

A sample of the algorithms generated is available at (Nadini 2020). The entire set of algorithms is available upon request.

## Abbreviations

SIR: susceptible–infected–removed
SIS: susceptible–infected–susceptible

## Footnotes

1 We consider that *N* is a multiple of *L*.

2 The distance between a point and a set is defined as the minimum Cartesian distance between the point and a generic point of the set.

3 The clustering coefficient is computed by considering a network that includes all the links that are generated during the entire observation window and counting the fraction of triadic closures (that is, whether two agents that are connected to a third one are also linked one another).

4 In order to allow to agents to exit from the base location, the probability to jump outside should be *p*>0.
